# A Study on the Genetics of Primary Ciliary Dyskinesia

**DOI:** 10.3390/jcm10215102

**Published:** 2021-10-30

**Authors:** Mohammed T. Alsamri, Amnah Alabdouli, Durdana Iram, Alia M. Alkalbani, Ayesha S. Almarzooqi, Abdul-Kader Souid, Ranjit Vijayan

**Affiliations:** 1 Department of Pediatrics, Tawam Hospital, Al Ain P.O. Box 15258, United Arab Emirates; malsamri@seha.ae (M.T.A.); asmabdouli@seha.ae (A.A.); diram@seha.ae (D.I.); alkalbani@seha.ae (A.M.A.); 2 Department of Pediatrics, College of Medicine and Health Sciences, United Arab Emirates University, Al Ain P.O. Box 17666, United Arab Emirates; 201707891@uaeu.ac.ae; 3 Department of Biology, College of Science, United Arab Emirates University, Al Ain P.O. Box 15551, United Arab Emirates; 4 Big Data Analytics Center, United Arab Emirates University, Al Ain P.O. Box 15551, United Arab Emirates

**Keywords:** genetic counseling, Arabian Peninsula, primary ciliary dyskinesia, respiratory infections, sinusitis, infertility, dextrocardia, situs inversus

## Abstract

Primary ciliary dyskinesia (PCD) is a poorly understood disorder. It is primarily autosomal recessive and is prevalent in tribal communities of the United Arab Emirates due to consanguineous marriages. This retrospective study aimed to assess the pathogenicity of the genetic variants of PCD in indigenous patients with significant clinical respiratory problems. Pathogenicity scores of variants obtained from the chart review were consolidated using the Ensembl Variant Effect Predictor. The multidimensional dataset of scores was clustered into three groups based on their pathogenicity. Sequence alignment and the Jensen–Shannon Divergence (JSD) were generated to evaluate the amino acid conservation at the site of the variation. One-hundred and twelve variants of 28 genes linked to PCD were identified in 66 patients. Twenty-two variants were double heterozygous, two triple heterozygous, and seven homozygous. Of the thirteen novel variants, two, c.11839 + 1G > A in dynein, axonemal, heavy chain 11 (*DNAH11*) and p.Lys92Trpfs in dynein, axonemal, intermediate chain 1 (DNAI1) were associated with dextrocardia with situs inversus, and one, p.Gly21Val in coiled-coil domain-containing protein 40 (CCDC40), with absent inner dynein arms. Homozygous *C1orf127*:p.Arg113Ter (rs558323413) was also associated with laterality defects in two related patients. The majority of variants were missense involving conserved residues with a median JSD score of 0.747. Homology models of two deleterious variants in the stalk of DNAH11, p.Gly3102Asp and p.Leu3127Arg, revealed structural importance of the conserved glycine and leucine. These results define potentially damaging PCD variants in the region. Future studies, however, are needed to fully comprehend the genetic underpinnings of PCD.

## 1. Introduction

Primary ciliary dyskinesia (PCD), also known as ‘motile ciliopathy’ or ‘motile ciliary dysfunction’, is a heterogeneous clinical entity, predominantly due to biallelic genetic variants. The disorder is associated with frequent respiratory infections, laterality defects (e.g., situs inversus in 50% of patients), and infertility (due to impaired functions of the Fallopian tubes and spermatozoa) [1,2]. Affected children typically have chronic or frequent respiratory complaints (e.g., rhinitis, otitis media, sinusitis, wet cough, wheezing, bronchitis, pneumonia, and bronchiectasis) from early infancy [3,4]. Its diagnosis is challenging, as variations in over 50 genes are known to be associated with PCD [5]. In addition, hundreds of proteins are involved in the axonemes, excluding the membrane-bound ones. Furthermore, some of the reported pathogenic variants are associated with normal nasal ciliary ultrastructure (e.g., those involving the dynein axonemal heavy chain 11 (*DNAH11*), coiled-coil domain-containing protein 65 (*CCDC65*), etc.) [6,7].

The axoneme contains an outer ring of nine-linked microtubular doublets, each possessing two motor complexes, the ‘outer dynein arm’ (composed of at least 16 distinct proteins) and the ‘inner dynein arm’ (composed of at least 12 subunits, with regulatory domains located within the ‘nexin’ linkage between doublets) [5]. The dynein arms contain ‘ATPases associated with diverse cellular activities’ (AAA-ATPase) proteins that power the beating cilia. Furthermore, a ‘central pair’ of single microtubules is tied to the outer ring by ‘radial spokes’. Many additional proteins are also essential for supporting and regulating the microtubular assembly (a ‘9 + 2 axoneme’) and beating [5]. Examples of dysfunctional gene groups include variants of coiled-coil domain-containing protein 39 (*CCDC39*) and coiled-coil domain-containing protein 39 (*CCDC40)*, which typically cause absent inner dynein arms with axonemal disorganization [8]. These genes encode molecular rulers (96-nm repeats along the axoneme), essential for the assembly of the nexin-dynein regulatory complex [9]. Thus, variants in *CCDC39* and *CCDC40* account for a significant number of PCD cases associated with axonemal disorganization [10,11]. Variants of *DNAH1* (dynein, axonemal, heavy chain 1), *DNAH5* (most frequently reported ones), *DNAI1* (dynein, axonemal, intermediate chain 1), and *DNAI2* usually cause defects confined to the outer dynein arm, resulting in impaired motility of the cilia. Variants of *ARMC4* (armadillo repeat-containing protein 4) typically impair the outer dynein arm docking, resulting also in absent outer dynein arms. Variants of *DNAAF1* (dynein, axonemal, assembly factor 1), *DNAAF2*, *DNAAF3*, *DNAAF5*, and *ZMYND10* (zinc finger MYND domain-containing protein 10) cause absent inner and outer dynein arms with encoded assembly factors remaining in the cytoplasm. In fact, variations in ciliary dyneins, primarily those involving *DNAH5*, *DNAH11,* and *DNAI1,* account for a large number of PCD cases [10,12]. Variants of *RSPH1* (radial spoke head component 1), *RSPH4A*, and *RSPH9* cause defects confined to the radial spoke or central apparatus with impaired assembly (e.g., off-center central pair and disorganized peripheral doublets). Variants of *HYDIN* (HYDIN axonemal central pair apparatus protein), on the other hand, may not reveal obvious structural anomalies on transmission electron microscopy. Variants of *DRC1* and *CCDC65* cause defects in the ‘nexin link dynein regulator complex proteins’ (N-DRCs).

In contrast to cystic fibrosis, diagnosing PCD is challenging due to the lack of a gold standard test [13]. Available structural and functional assays have limited accessibility and high-cost [14]. For example, examination of the ciliary structure by transmission electron microscopy (TEM) requires skills in sample collection, processing, and interpretation, and normal findings have been reported in genetically confirmed cases [15]. Immunofluorescence labeling of specific ciliary proteins is highly specific, but requires other confirmatory investigations [13,16]. High-speed video microscopy has been used to assess ciliary beat pattern and frequency; this analytical tool, however, is not yet available in our region [4]. Nasal nitric oxide is reduced in most patients with PCD [17]; this promising test, however, requires co-operative patients, typically children five years or older. Notably, normal nasal nitric oxide have been described in variants involving the radial spoke (e.g., *RSPH1*) [18]. Genetic testing, using either available panels or whole genome/exome sequencing, requires a simple blood collection and may offer an alternative approach to investigating PCD [19].

To date, the treatment of PCD is only supportive (proper vaccination, minimizing exposure to respiratory pathogens, good nutrition, antibiotics for bacterial infections, and mucolytic agents) [20]. The disease, however, is amenable to ‘prevention’ through genetic screening and counselling, especially in cultures that practice frequent consanguineous marriages [21]. Therefore, it is essential to analyze PCD variants in communities and select clinically relevant ones for screening and counselling.

Emirati people, about one million [22], have tribal heritages that include Arabs, Persians, Baluchis, and East Africans. Founder mutations and autosomal recessive disorders are exceptionally common in the UAE due to consanguineous marriages [23]. The genetics of PCD in this population have been reviewed [24,25,26,27,28,29]. Higher prevalence has been reported among Arabs [5], including variants found in the United Arab Emirates (UAE), such as those involving the *RSPH9* and *RSPH4A* genes [27,29]. Nevertheless, research involving PCD in the Arab world is still limited [26], and further population studies are highly justified. This report assesses the pathogenicity of variants in PCD-related genes found in native Emirati children with clinically significant respiratory problems.

## 2. Methods

The pediatric pulmonary service at Tawam Hospital, Al Ain, UAE is a tertiary center that serves children with respiratory problems. This study was a retrospective review of the genetic investigations performed at Tawam Hospital. It analyzed the results of the genetic tests—chromosomal microarray, comprehensive pulmonary disease panel (includes the entire coding plus 10 base pairs of the flanking intron regions of 92 single genes, see Supplementary Table S1, https://www.centogene.com/science/centopedia/comprehensive-pulmonary-disease-panel.html, accessed on 17 September 2021), single-gene sequencing, and diagnostic WES—of 66 pediatric patients with chronic or frequent respiratory infections and positive variants in PCD genes. The PCD-related genes included in the Panel were: *CCDC39, CCDC40, DNAAF1, DNAAF2, DNAH11, DNAH5, DNAH9, DNAI1, DNAI2, DNAL1, NME8, RSPH1, RSPH4A,* and *RSPH9*.

The study was approved by ‘Tawam Human Research Ethics Committee’ (T-HREC); reference numbers: SA/AJ/566 (19 April 2018 and 11 December 2019) and AA/AJ/653 (19 June 2019). Informed consent to participate in this retrospective data collection for the reported variants was exempted. All methods were performed in accordance with the relevant guidelines and regulations. The data were collected from 2013 to 2019; 120 patients had genetic investigations, and 66 had positive results.

The pediatric pulmonary service at Tawam Hospital receives over four thousand outpatient visits per year. Genetic testing is usually performed for children with chronic or recurrent unexplained and clinically significant respiratory problems. Typically, these children undergo work-up that includes sweat chloride test, chest computed tomography (CT) scan, and gastrointestinal studies. When necessary, the pediatric pulmonary team will also request a comprehensive pulmonary disease panel or targeted mutation (when known disease-causing variant is present in the family). If this investigation is negative or a genetic condition is suspected, WES will be then requested by the genetic team, usually with chromosomal microarray (CMA) or duplication/deletion studies. The variants reported here include positive reports found in the comprehensive pulmonary disease panel (*n* = 48), WES (*n* = 16), and/or single gene sequencing (*n* = 8); see Table 1 and Supplementary Table S2. In addition, chromosomal microarray (*n* = 15), duplication/deletion (*n* = 5; results were negative), and other specific tests (e.g., the nasal scrape biopsies performed in two children) are also included. It is worth emphasizing that the 66 children studied had at least the comprehensive pulmonary disease panel, WES, or single gene sequencing. Eight children had both the comprehensive pulmonary disease panel and WES; the outcome of these tests is also summarized in Table 1 and Supplementary Table S2. Due to hospital arrangements, many of these tests were performed by Centogene AG (Germany). The reported WES coverage was 99.40%, and variants were confirmed by Sanger sequencing. Variants of HYDIN were also verified by Sanger sequencing, but primer designs were not available to distinguish between HYDIN and the pseudogene HYDIN2. The term ‘double heterozygous’ was used here since, in most patients, parental data were not available to phase the variants.

### 2.1. Information from Public Databases

Variant information, available in public databases, was consolidated using Ensembl Variant Effect Predictor (VEP; https://www.ensembl.org/Tools/VEP; Accessed on 3 February 2020) [30]. Extracted data included effect of variation, codon and amino acid changes (where applicable), known variations from dbSNP (Single Nucleotide Polymorphism Database), functional consequence, exome allele frequency from gnomAD, splicing prediction from SpliceAI, as well as score and pathogenicity prediction from the algorithms ranked REVEL (rare exome variant ensemble learner), ranked MetaLR (meta-analytic logistic regression), ranked MetaSVM (meta-analytic support vector machine), Condel (consensus deleterious) and scaled CADD (combined annotation-dependent depletion) available in dbNSFP (One-Stop Database of Functional Predictions and Annotations for Human Non-synonymous and Splice Site) version 4.0a. Clinical assessments included only those with functional evidence supportive. ACMG (American College of Medical Genetics and Genomics) classification was obtained from https://www.varsome.com (Accessed on 3 February 2020).

### 2.2. Multiple Sequence Alignment

Multiple sequence alignment was performed to evaluate conservation of amino acids at the sites reported here and to compute Jensen–Shannon Divergence (JSD) scores. Amino acid sequences, where available, of proteins from *Homo sapiens* (human), *Pan troglodytes* (chimpanzee), *Mus musculus* (house mouse), *Rattus norvegicus* (Norway rat), *Canis lupus familiaris* (dog), *Equus caballus* (horse), *Bos taurus* (bovine), *Xenopus tropicalis* (frog), *Gallus gallus* (chicken), and *Danio rerio* (zebrafish) were downloaded from NCBI RefSeq (Supplementary Table S3). Sequences were imported into Geneious 9.1.8 (Biomatters Ltd., Auckland, New Zealand) and multiple sequence alignment was performed using MUSCLE [31]. Aligned sequences were exported in FASTA format.

### 2.3. Jensen–Shannon Divergence (JSD)

Using multiple sequence alignment of the sequences from the species mentioned above, conservation of protein residue at the sites reported here was predicted using the JSD method [32]. This task was performed using the online tool available at https://compbio.cs.princeton.edu/conservation/score.html (Accessed on 3 December 2019). The default settings were used for this purpose.

### 2.4. Clustering and Multidimensional Scaling

**Figure 1:**
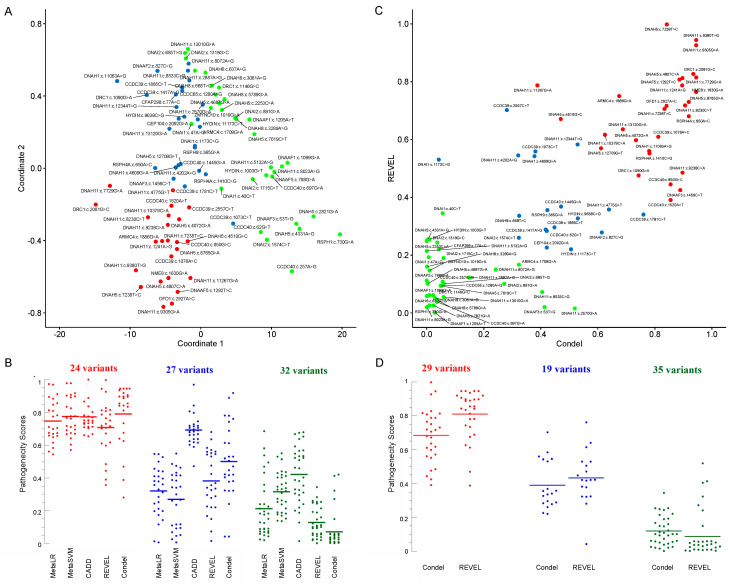
(**A**) Multidimensional scaling (MDS) plot of MetaLR, MetaSVM, CADD, REVEL, and Condel scores. Here, MDS reduces the dimensionality of the data and projects it on two abstract coordinates, which visualize the ‘closeness’ of points. The points were clustered into three groups using k-means clustering and colored red, blue, and green. The differences between the three groups (red, blue, and green) for each prediction scoring tool are significant (*p* < 0.0001, Kruskal–Wallis H test). (**B**) A dot plot of the five pathogenicity predictor scores arranged according to the clusters identified in (**A**); the CADD scores were divided by 33 to aid data comparison. Horizontal lines are mean. (**C**) A scatter plot of REVEL versus Condel scores of the missense variants. The three k-means clusters obtained are colored in red (likely pathogenic), blue (uncertain), and green (likely benign). The difference between the clusters was significant (*p* < 0.0001, Kruskal–Wallis H test). (**D**) A dot plot of the five pathogenicity predictor scores arranged according to the clusters identified in (**C**). Horizontal lines are mean.

The variants were classified into three clusters—possibly pathogenic, uncertain, and possibly benign—by k-means clustering using the k-means function in R version 3.6.0, using a combination of predictors (the pathogenicity predictions from all algorithms mentioned in 2.1). The clustering methods yielded *p* values of <0.0001 on the Kruskal–Wallis test between the three groups (possibly pathogenic, uncertain, and possibly benign) for each of the scoring systems (see Figure 1 and Table 2). To reduce the dimensionality of the dataset, multidimensional scaling (MDS) was performed with the pathogenicity scores using the cmdscale function in R.

### 2.5. Structural Modeling

Homology models of two deleterious variants in the stalk region of DNAH11, p.Gly3102Asp, and p.Leu3127Arg were generated. For comparative modeling, the stalk region of the human cytoplasmic dynein 1 (Protein Data Bank, PDB, ID: 5NUG) structure was used. Sequences of the stalk region were aligned, and models of the wild-type (WT) and the two variants were generated using Schrödinger Prime 2019-4 (Schrödinger, LLC, New York, NY, USA).

### 2.6. Statistics

The analyses (including means and standard deviations) were performed using SPSS statistical package (Version 20; IBM Corp., Armonk, NY, USA). The Kruskal–Wallis H test (non-parametric, k independent samples) test was used to compare groups of variants. A *p* value of <0.05 was considered significant.

## 3. Results

One hundred and twelve variants of 28 PCD genes were found in 66 patients. Table 2 summarizes the results of the 112 studied variants of PCD; these variants were found in the 66 pediatric patients with chronic or frequent respiratory infections and positive variants in PCD genes. Table 3 lists the 18 patients with ‘double or triple heterozygous variants’ or ‘homozygous variants’ in PCD genes.

Eighty-three variants were missense, thirteen intronic (splice site), four nonsense, four frameshift, six synonymous, one duplication, and one inframe deletion. The majority of the missense variants involved conserved residues with a JSD score (mean ± SD) of 0.724 ± 0.102 (median, 0.747).

An MDS plot (Figure 1A) of the five scores (MetaLR, MetaSVM, CADD, REVEL, and Condel; see Table 2) revealed 24 variants clustered in the left lower zone (‘red’). As examples, their Condel scores were 0.790 ± 0.186 (median, 0.855) and CADD scores 25.5 ± 3.2 (median, 24.7) (Figure 1B). Thirty-two variants clustered in the right upper lower zone (‘green’). Their Condel scores were 0.070 ± 0.117 (median, 0.029) and CADD scores 13.7 ± 5.8 (median, 13.3), Figure 1B. The remaining 27 variants were centrally located and colored blue. Their Condel scores were 0.500 ± 0.225 (median, 0.471) and CADD scores 22.8 ± 3.0 (median, 22.8), Figure 1B.

A scatter plot of REVEL versus Condel scores of the missense variants was also clustered into three groups (Figure 1C and Table 2). Twenty-nine clustered in the right upper zone. Their Condel scores were 0.683 ± 0.157 (median, 0.706) and REVEL scores 0.809 ± 0.143 (median, 0.855) (Figure 1D); they were considered ‘possibly pathogenic’ (‘red’). Thirty-five variants clustered in the left lower zone. Their Condel scores were 0.119 ± 0.086 (median, 0.101) and REVEL scores 0.086 ± 0.136 (median, 0.037). These variants were considered ‘possibly benign’ (‘green’). The remaining 19 variants were centrally located. Their Condel scores were 0.389 ± 0.141 (median, 0.340) and REVEL scores 0.433 ± 0.154 (median, 0.421). These variants were considered ‘uncertain’ (‘blue’).

**Figure 2:**
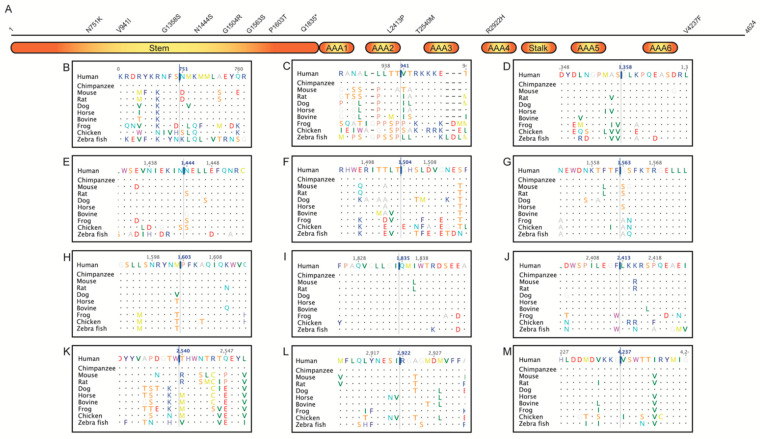
(**A**) Schematic representation of the DNAH5 protein (4624 amino acids). Stem, stalk, and ATPases Associated with a variety of cellular Activities (AAA) regions are shown as blocks. Missense and nonsense variations identified here are displayed above the block diagram. Twenty-one amino acid regions, centered around missense/nonsense variations, obtained from a multiple sequence alignment of DNAH5 proteins from human, chimpanzee, mouse, rat, dog, horse, bovine, frog, chicken, and zebra fish are shown in B-M–(**B**) N751K (c.2253C>A); (**C**) V941I (c.2821G>A); (**D**) G1358S (c.4072G>A); (**E**) N1444S (c.4331A>G); (**F**) G1504R (c.4510G>C); (**G**) G1563S (c.4687G>A); (**H**) P1603T (c.4807C>A); (**I**) Q1835Ter (c.5503C>T); (**J**) L2413P (c.7238T>C); (**K**) T2540M (c.7619C>T); (**L**) R2922H (c.8765G>A); (**M**) V4237F (c.12709G>T).

Variants in DNAH5 (19 of 112) and DNAH11 (21 of 112) were the greatest numbers identified here. Eight variations of DNAH5 were in the stem, one in the AAA2 (ATPases associated with a variety of cellular activities 2), one between the AAA2 and AAA3, one in the AAA4, and one in the C-terminus (Figure 2). Six of the missense variations involved highly conserved residues (Table 2).

**Figure 3:**
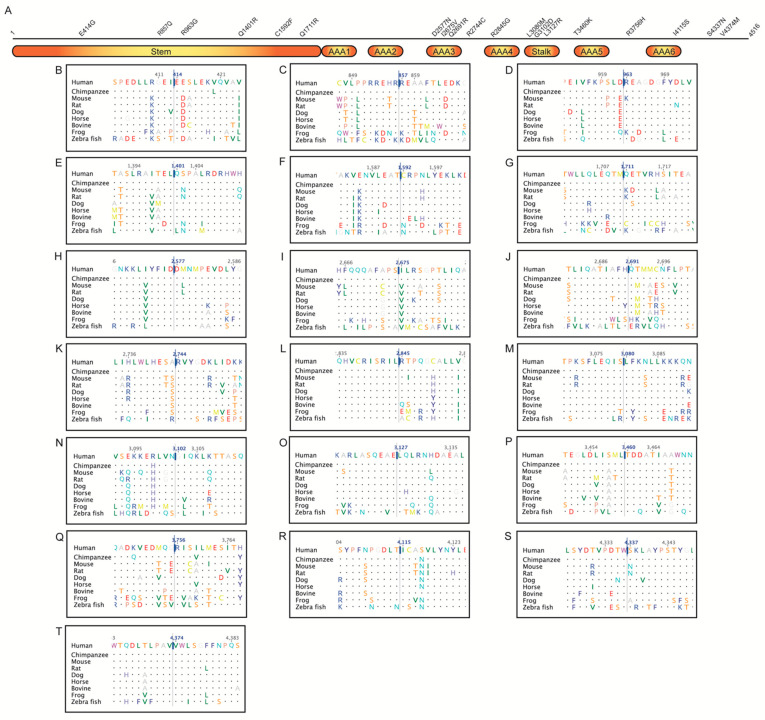
(**A**) Schematic representation of the DNAH11 protein (4516 amino acids). Stem, stalk, and ATPases Associated with a variety of cellular Activities (AAA) regions are shown as blocks. Missense and nonsense variations identified here are displayed above the block diagram. Twenty-one amino acid regions, centered around missense/nonsense variations, obtained from a multiple sequence alignment of DNAH11 proteins from human, chimpanzee, mouse, rat, dog, horse, bovine, frog, and zebra fish are shown in B–T–(**B**) E414G (c.1241A>G); (**C**) R857Q (c.2570G>A); (**D**) R963G (c.2887A>G); (**E**) Q1401R (c.4202A>G); (**F**) C1592F (c.4775G>T); (**G**) Q1711R (c.5132A>G); (**H**) D2577N (c.7729G>A); (**I**) I2675V (c.8023A>G); (**J**) Q2691R (c.8072A>G); (**K**) R2744C (c.8230C>T); (**L**) R2845G (c.8533C>G); (**M**) L3080M (c.9238C>A); (**N**) G3102D (c.9305G>A); (**O**) L3127R (c.9380T>G); (**P**) p.Thr3460Lys (c.10379C>A); (**Q**) R3756H (c.11267G>A); (**R**) I4115S (c.12344T>G); (**S**) S4337N (c.13010G>A); (**T**) V4374M (c.13120G>A).

**Figure 4:**
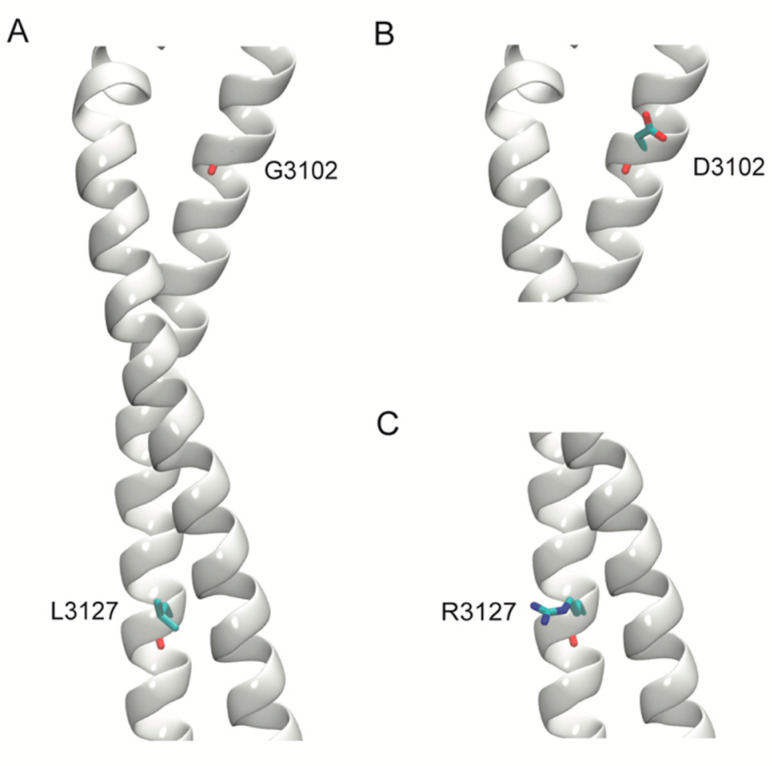
Models of the stalk region of the wild-type (WT) and variant structures of DNAH11. The stalk region of the protein is represented as a white helix and amino acids are shown in stick representation. (**A**) WT structure with G3102 and L3127. (**B**) Structure of the variant G3102D. (**C**) Structure of the variant L3127R.

Six variations of DNAH11 were in the stem, three in the AAA3, one between the AAA3 and AAA4, one in the AAA4, three in the stalk, one in the AAA5, one between the AAA5 and AAA6, one in the AAA6, and two in the C-terminus (Figure 3). The majority involved highly conserved residues (Table 2). Homology models of the two deleterious variants, Gly3102Asp and Leu3127Arg, in the stalk region of DNAH11 showed that these amino acids are also conserved in the homologous cytoplasmic dynein that was used for the modeling (Figure 4). In Gly3102Asp, the conserved achiral glycine gives flexibility to the helical structure and the aspartate is likely to disrupt this flexibility with its bulkier negatively charged sidechain. In Leu3127Arg, the hydrophobic leucine keeps the stalk intact, and the charged arginine is likely to disrupt this assembly.

Unfortunately, lack of suitable structures and missing regions in homologous structures limit modeling other variants. Structural information for the regions of interest in the other proteins is also not available. Therefore, further analysis of their missense variations is limited to information obtained from the multiple sequence alignment of proteins from various species (Supplementary Figures S1–S5). Selected variants with high or conflicting prediction scores are discussed below.

The three variations of DNAH1 involved conserved residues (Figure S1A–C) and had high pathogenicity scores. DNAH6 Ile213 was conserved in the 10 species considered (Figure S1D), and its substitution with valine was benign. DNAH8 Ile223 showed permissible valine substitutions (Figure S1E), which could support CADD score (22.7) for the Ile223Thr variant (Table 2).

CCDC39 Lys359 was conserved (Figure S2B), which could support the pathogenic scores of MetaLR, CADD, and Condel for Lys359Thr. CCDC39 Asn473 was also conserved (Figure S2C), which could support CADD score (25.5) for Asn473Asp. CCDC39 Thr594 was not conserved (Figure S2D), which supported CADD (11.1) and REVEL (0.341) scores for Thr594Ile (Table 2).

CCDC40 Gly21 was substituted only by aspartate or glutamate in four of the nine studied species (Figure S2H); phenotypically, the novel Gly21Val is probably pathogenic (Table 3, Patient 8) despite its benign computational pathogenicity predictions (Table 2). CCDC40 Asp284 resided in a region where the sequence was conserved and a conservative substitution to a glutamate was observed in only two no-mammalian species (chicken and zebrafish) studied here (Figure S2K); thus, histidine might not be tolerated at this position. Asp284His, on the other hand, had conflicting predictions of pathogenicity (Table 2). This variant was detected in a double heterozygous state in a symptomatic child with recurrent sinusitis (Table 3, Patient 7). Thus, future studies are needed to confirm whether this variant is disease causing.

DNAAF1 Glu402 was substituted by isoleucine in zebrafish (Figure S3B). This might support the benign scores for Glu402Val, as valine could usually substitute for isoleucine. DNAI2 Ser229 was conserved (Figure S4D), which might support CADD score (21.1) for Ser229Ala. DRC1 Arg694 was conserved (Figure S5C), which might support the pathogenicity of Arg694Thr. HYDIN Pro3213 was substituted by different amino acids in two species (Figure S5E); the novel variant Pro3213Arg had conflicting predictions of pathogenicity (Table 2), but it was clinically likely pathogenic (Table 3, Patients 17–18). RSPH4A Tyr217 was conserved (Figure S5H), which supported the pathogenic scores of Condel and CADD for Tyr217Ser. RSPH4A Ile470 was also conserved except for the valine substitution (Figure S5I), which supported the deleterious Condel score (0.781) for Ile470Met. ARMC4 Arg570 was conserved (Figure S5K), which supported the CADD score (20.3) for Arg570Gln. ARMC4 Arg629 was conserved (Figure S5L), which supported Condel and CADD scores for Arg629His. CEP104 Glu698 was conserved (Figure S5M), which might support CADD score (23.3) for the novel variant Glu698Lys (Table 2 and Table 3, Patient 9).

C1orf127 Arg113Ter was found in homozygous state in two related children (double cousins) with complex congenital heart disease and lateralization defect. The parents were heterozygous and asymptomatic (had situs solitus on chest radiographs and echocardiograms), suggesting autosomal recessive inheritance. Further studies are needed to understand the precise function of C1orf127 protein.

### Novel Variants

Thirteen variants were novel, mostly identified in symptomatic children (Table 3). Eight of the novel variants were missense, two frameshift, one nonsense, one duplication, and one splice donor site (Table 2). CCDC40:p.Gly21Val was found in homozygous state in two symptomatic sisters with biopsy-proven significant microtubular disorganizations, including distorted dynein arms and absent inner dynein arms, thus, confirming its pathogenicity (Table 3, Patient 8). It is worth noting that CCDC40 Gly21 was not conserved (Figure S2H), which accounts for the relatively low Condel and CADD scores (Table 2).

DNAH5:p.Leu2413Pro (Condel: 0.842, CADD: 29.2) was found in a heterozygous state with heterozygous DNAAF5:p.Arg263Gln (Condel: 0) in a child with significant respiratory infections from early infancy. DNAH5 Leu2413 was conserved (Figure 2J). Consistently, the scores (including MDS) of DNAH5:p.Leu2413Pro predict pathogenicity. This variant was detected in a child with significant respiratory infections from early infancy. Therefore, other unidentified conditions could be responsible for the observed phenotype in this patient. DRC1:p.Glu382Asp had a Condel score of 0.022, which was consistent with the presence of aspartate at this position in various species (Figure S5B).

DNAH11:p.Glu414Gly was found in heterozygous state with two other heterozygous variants (CCDC39:p.Arg853Cys and DNAAF3:p.Ile18Ser) in a child with multiple anomalies. Its high Condel (0.896) and CADD (24.2) scores were consistent with the conserved DNAH11 Glu414 (Figure 3B). DNAH11:p.Leu3080Met involved the highly conserved DNAH11 Leu3080 (Figure 3M), supporting its Condel score of 0.987. It was found in a symptomatic child with heterozygous STAT3:p.Met660Thr, known to cause ‘hyper-IgE recurrent infection syndrome 1, autosomal dominant’. Thus, future studies are needed to determine whether these variants are disease causing or not. DNAH11:c.11839+1G>A, with potential loss of splice donor site predicted by SpliceAI (donor loss delta score: 1; i.e., could be pathogenic), was found in a double heterozygous state with DNAH11:p.Arg2744Cys (Condel: 0.906) in a child with situs inversus/dextrocardia, suggesting pathogenicity (Table 3, Patient 15).

DNAI1:p.Lys92Trpfs was found in a heterozygous state with the X-linked recessive OFD1:p.Lys976Thr. OFD1 is linked to ciliary dysfunction (Joubert syndrome 10, X-linked recessive). ACMG classification from VarSome for OFD1:p.Lys976Thr is uncertain significance, but computational predictors indicate that it is ‘possibly damaging’ (Table 2). The affected girl had situs inversus and chronic respiratory infections, likely resulting from X-inactivation.

The remaining novel variants were: CCDC39:p.Glu598* (CADD, 37); CCDC40:c.1097delT (frameshift); CEP104:p.Glu698Lys (Joubert syndrome 25, Figure S5M, Table 3, Patient 9); DNAAF5:p.Val431Ala (Condel: 0.886, Figure S3F); DNAH5 duplication of exon 1–48 (unknown significance); HYDIN:p.Pro3213Arg (likely pathogenic, Figure S5E, Table 3, Patients 17–18).

## 4. Discussion

Here, we analyzed the pathogenicity of genetic variants of PCD genes in the UAE. Two noticeable findings are the novel pathogenic variants of PCD in the community, and the clustering of up to seven distinct variants in some patients. High rates of inbreeding are known to increase the genomic homozygosity past prediction [33].

No individual or meta predictor is able to clearly predict the pathogenicity of all clinically relevant variants [34]. MDS is a statistical approach adopted in the interpretation of datasets involving several variables (ttp://www.stat.yale.edu/~lc436/papers/JCGS-mds.pdf; accessed on 25 October 2021). Hence, here we employed MDS to segregate and cluster variants based on multiple pathogenicity scores. As evident from Figure 1A, the reduction in dimensionality into two coordinates, while still preserving the distance between these points in multi-dimensional space, facilitated better visualization and separation of these variants into three groups. These groups were designated as likely pathogenic, uncertain, or likely benign. Overlaps, however, were observed between the range of individual predictors (Figure 1B). Figure 1B also indicated that the meta predictors REVEL and Condel were able to segregate the three categories in this dataset. Thus, a scatter plot was generated based on REVEL and Condel scores (Figure 1C), where the three groups clearly cluster separately (Figure 1D). In the absence of clear evidence of the pathogenicity of variants (e.g., functional assays), such analysis could assist with inferring potential pathogenicity of variants. Thus, care must be taken while interpreting in silico pathogenicity scores in isolation.

The occurrence of one or more autosomal recessive disorders of PCD in the offspring of a couple is a function of the number of shared variants. This probability is estimated by binomial distribution, which shows a 0.4375 chance for two shared variants and a 0.8665 chance for seven shared variants. Thus, although the incidence rate in the UAE is unknown, PCD appears to be frequent in the community and needs preventive measures.

It is worth noting that *DNAH1*:p.Tyr3688Cys (as an example) was found in heterozygous state in a child with dextrocardia; the only other variant reported in the genes of interest in this patient was *DNAH6*:p.Ile213Val (Condel: 0.00). Other unidentified recessive conditions (not reported or detected by the WES test) could be responsible for the observed phenotype in this patient. Future studies are needed to ratify the inheritance of some of these variants, as autosomal recessive diseases are more amenable to genetic prevention than autosomal dominant ones.

As explained in the Results, *CCDC40*:p.Gly21Val (Table 3, Patient 8) is identified in homozygous state in two siblings with biopsy-proven significant microtubular disorganizations, including distorted dynein arms and absent inner dynein arms, confirming its pathogenicity. The computational variant effect predictions, however, are ‘benign’ (‘tolerated’). This discrepancy in ‘in silico prediction’ is serious, especially with respect to genetic screening. In addition, many variants may be missed or incorrectly called by genome sequencing approaches. Thus, future studies are also needed to further investigate the pathogenicity of some of these variants (e.g., parental studies to help evaluate effects of single heterozygous variants) and improve the yield of genetic investigations.

Many of the PCD variants were found in children with other congenital anomalies and developmental delay (e.g., Patients 1, 2, and 9 in Table 3). Unidentified variations in other genes, thus, may have also contributed to the overall phenotype, given the high incidence of recessive disorders in our population. Future studies, thus, are needed to uncover all contributing genetic variants to the disease in any given patient. Exome sequencing study was performed on individuals with PCD from Saudi Arabia [24]. The study identified similar variants in *CCDC39*, *CCDC40*, *DNAAF5*, *DNAH5*, *DNAI1*, *RSPH4A*, and *RSPH9*. The study also identified other PCD variants that involved genes not in the present cohort, such as: *PKD1L1*, *MCIDAS*, *CCDC151*, *CCNO*, *CYP21A2*, *ITCH*, *MCIDAS*, and *CEP164* [24].

The majority of the variants reported here are heterozygous and are found with other multiple heterozygous variants in symptomatic children. Further studies are needed to uncover whether individuals with heterozygous variants in PCD genes could be symptomatic, especially with genes that may show monoallelic expression [35,36]. Undoubtedly, the observed PCD alleles could result in complex phenotypes, as shown in Table 3. As recently shown, the immunofluorescence analysis is a reliable diagnostic tool for PCD, and could be used to fulfill this purpose [13].

Genetic variations from Arabian populations are underrepresented in publicly databases, such as ClinVar and Genome Aggregation Database (gnomAD). Often, this shortage impacts the ability of clinicians and genetic counsellors to draw meaningful conclusions about the variants identified. Thus, studies that catalogue variations in the community are of importance, especially in regions of a high rate of consanguinity that potentially exacerbates the prevalence of recessive disorders. This holds true for PCD, where the diagnosis still remains challenging [26]. Thus, this study describes the variations in PCD-related genes identified through a retrospective chart review of patients at a tertiary hospital in the UAE. Based on our current practice and commercial sequencing arrangement, most of the variants were identified from a comprehensive pulmonary disease panel, which covers only 14 (Supplementary Table S1) of the over 45 genes associated with PCD [10,37].

Hence, it is possible that other variations in PCD-related genes could have been overlooked. Despite this limitation, 112 variations, 13 of which are novel, have been identified in this study. Nonetheless, when PCD (ciliopathy) is suspected, it is prudent to use a panel that covers all the genes known to be associated with this condition. As 25% of the described variations in this study have been designated as ‘variants of uncertain significance’ in the publicly databases, it underscores the need for further studies to elucidate their role in PCD. Additionally, studies involving parents and siblings could also assist phasing and establishing the role of double and triple heterozygous variants identified here. Large-scale population-based studies using WES- or PCD-specific panels may be necessary to provide a comprehensive coverage of variations that underpin PCD this disease, and potentially guide future genetic screening programs and premarital counselling.

This investigation was retrospective and aimed at assessing variants identified in the community. Ideally, we would have preferred additional functional studies. Therefore, future investigations should aim at further characterization of these variants, including functional analyses.

## 5. Conclusions

The clinical spectrum of PCD is far from being well understood. Therefore, reports on variants associated with the disease are highly desirable. This condition is especially common in the Arabian Peninsula [24]. Our results describe several damaging variants in PCD genes in various Arabian tribes residing in the UAE. Considering the small local population of the UAE (about one million), these results could support genetic screening and counseling programs to prevent the disease. More work, however, is needed to understand the scope of the genetic underpinnings of PCD. Further studies addressing the limitations and methods of improving in silico analysis of genetic variants (e.g., including parental and sibling studies) are also necessary. Methods that analyze variants within the structure of a functioning cilium are warranted. As previously stated, PCD variants are distinctive to families, and healthcare providers need to be familiar with their significance in the community [24].

## Figures and Tables

**Table 1:** Investigations performed in the studied children. Supplementary Table S2 describes the investigation of each child ^(1)^.

	Comprehensive Pulmonary Disease Panel	Whole (Diagnostic) Exome Sequencing (WES)	Chromosomal Microarray (CMA)	Targeted Deletion/Duplication Analysis	Single Gene Sequencing
Tests done	48	16	15	5	8
PCD genetic defect detected	48	13	5	0	8
PCD genetic defect not detected	0	3 (Three children had negative WES; thereafter, a variant was detected by the Panel in two of them (Patients 47 and 48; Supplementary Table S2) and duplication in exons 1–47 of DNAH5 was detected by CMA in another child (Patient 50)).	10	5	0

^(1)^ Six children had both the Panel and WES, the outcome of these tests was as follows: Patients 47 and 48 had negative WES; the Panel detected variants in PCD-related genes. In four Patients (9, 19, 26, and 58), both tests detected variants in PCD-related genes. For Patients 19 and 26, in each the same variants in PCD-related genes were detected by the Panel and WES. For Patients 9 and 58, each different variant in PCD-related genes were detected by the Panel and WES.

**Table 2:** Studied variants of PCD. Exome allele frequency is from gnomAD; * indicates if homozygotes have been reported. JSD (Jensen–Shannon Divergence) scores range from 0 to 1.0, with higher values indicating better conservation; ‘Conserved’ refers to complete conservation of the amino acid at the site over the 10 studied species (see Methods) and ‘figure numbers’ to the multiple sequence alignment. MetaLR (*meta*-analytic logistic regression that integrates variant pathogenicity scores and allele frequency to predict deleteriousness) ranked scores range from 0 to 1; higher scores are a more likelihood of pathogenicity. MetaSVM (*meta*-analytic support vector machine that integrates multiple omics data) ranked scores range from 0 to 1; higher scores are a more likelihood of pathogenicity. CADD (combined annotation-dependent depletion) refers to PHRED-scaled CADD scores that range from 1 to 99 (e.g., a score of 30 suggests the variant is top 0.1%). REVEL (rare exome variant ensemble learner that integrates data from several pathogenicity predictor software) ranked scores range from 0 to 1; higher scores are a more likelihood of pathogenicity. Condel (consensus deleterious generated from SIFT (made from sequence homology and biochemistry of the alternate residue) and PolyPhen2 (made from sequence homology and known database of the protein secondary structure)) scores range from 0.0 (tolerated) to 1.0 (deleterious). ‘Scatter’ refers to k-means clusters in the scatter plot of REVEL versus Condel scores (see Figure 1C) and ‘MDS’ refers to k-means clusters in the MDS plot of MetaLR, MetaSVM, CADD, REVEL, and Condel scores (see Figure 1A); red indicates possibly pathogenic, blue uncertain, and green possibly benign. American College of Medical Genetics and Genomics (ACMG) classification was from https://www.varsome.com (Accessed on 29 November 2020). Clinical assessment is for variants with supportive functional evidence. Patient phenotypes are summarized in Table 3. D, deleterious; T, tolerated; N, neutral.

Variant	Frequency	JSD	MetaLR	MetaSVM	CADD	REVEL	Condel	Scatter	MDS	ACMG	Clinical Assessment
*ARMC4*(NM_018076.4):c.1709G>A [cGg/cAg] (p.Arg570Gln) Missense, rs140569195	0.0004742	0.82265 Conserved Figure S5K	0.41008 T	0.26729 T	20.3	0.16698	0.323 N	Green	Blue	Likely benign	-
*ARMC4*(NM_018076.4):c.1886G>A [cGc/cAc] (p.Arg629His) Missense, rs200127444	0.00007571 *	0.82467 Conserved Figure S5L	0.774 T	0.7778 T	25.6	0.75005	0.675 D	Red	Red	Likely benign	-
*C1orf127*(NM_001170754.1):c.337C>T [Cga/Tga] (p.Arg113Ter) Nonsense, rs558323413	0.00002156	0.80196 Conserved Figure S5Q	-	-	28.1	-	-	-	-	Uncertain significance	Patient 2
*CCDC39*(NM_181426.2):c.1073C>T [aCa/aTa] (p.Thr358Ile) Missense, rs183413880	0.003822 *	0.63091 Figure S2A	0.44228 T	0.41582 T	15.58	0.55883	0.381 N	Blue	Blue	Benign	Patient 3
*CCDC39*(NM_181426):c.1076A>C [aAa/aCa] (p.Lys359Thr) Missense, rs956532574	-	0.7388 Conserved Figure S2B	0.81295 D	0.77653 T	24.3	0.60952	0.812 D	Red	Red	Uncertain significance	Patient 3
*CCDC39*(NM_181426.1):c.1167+1261A>G, Splice donor, rs577069249	-	-	-	-	-	-	-	-	-	Uncertain significance	Pathogenic, Patient 4
*CCDC39*(NM_181426.1):c.1363-3delC, Splice acceptor, rs551191744	-	-	-	-	-	-	-	-	-	Uncertain significance	Patient 5
*CCDC39*(NM_181426.1):c.1417A>G [Aat/Gat] (p.Asn473Asp) Missense, rs1241950069	0.00000811	0.81432 Conserved Figure S2C	0.28445 T	0.06946 T	22.8	0.28867	0.417 N	Blue	Blue	Likely benign	-
*CCDC39*(NM_181426.1):c.1528-11_1528-10delCT; Splice acceptor, rs765966793	0.00005236	-	-	-	-	-	-	-	-	Likely benign	-
*CCDC39*(NM_181426.2):c.1781C>T [aCa/aTa] (p.Thr594Ile) Missense, rs140505857	0.0006629	0.73478 Figure S2D	0.5455 T	0.41927 T	22.4	0.34042	0.760 D	Blue	Blue	Likely benign	Patient 5
*CCDC39*(NM_181426.1):c.1792G>T [Gag/Tag] (p.Glu598Ter) Nonsense, Novel	-	0.79808 Figure S2E	-	-	37	-	-	-	-	-	-
*CCDC39*(NM_181426.1):c.1885C>T [Cgc/Tgc] (p.Arg629Cys) Missense, rs199526690	0.0001162	0.77558 Figure S2F	0.198 T	0.09238 T	22.8	0.29614	0.448 N	Blue	Blue	Likely benign	-
*CCDC39*(NM_181426.2):c.2557C>T [Cgt/Tgt] (p.Arg853Cys) Missense, rs201097154	0.0003535	0.60342 Figure S2G	0.65899 T	0.57055 T	21.8	0.70179	0.281 N	Blue	Red	Likely benign	-
*CCDC39*(NM_181426.1):c.2660dupT [cta/ctTa] (p.Ser888fs) Frameshift, rs200353947	0.007916 *	-	-	-	-	-	-	-	-	Benign	-
*CCDC40*(NM_017950.3):c.62G>T [gGa/gTa] (p.Gly21Val) Missense, Novel	-	0.48751 Figure S2H	0.43298 T	0.34655 T	11.66	0.28361	0.421 N	Blue	Green	-	Pathogenic, Patient 8
*CCDC40*(NM_017950.3):c.257A>G [tAt/tGt] (p.Tyr86Cys) Missense, rs202220442	0.0000728	0.41972 Figure S2I	0.68522 T	0.55015 T	7.262	0.10822	0.056 N	Green	Green	Likely benign	Pathogenic, Patient 6
*CCDC40*(NM_017950.3):c.697G>A [Gat/Aat] (p.Asp233Asn Missense, rs201815496	0.0002182	0.47098 Figure S2J	0.2398 T	0.36059 T	10.07	0.0383	0.037 N	Green	Green	Likely benign	Patient 7
*CCDC40*(NM_017950.3):c.850G>C [Gac/Cac] (p.Asp284His) Missense, rs201042940	0.002833 *	0.79987 Figure S2K	0.71147 T	0.76216 T	23.5	0.44471	0.855 D	Red	Red	Benign	Likely pathogenic or uncertain significance, Patient 7
*CCDC40*(NM_017950.3):c.1097delT [cTg/cg], Frameshift, Novel	-	-	-	-	-	-	-	-	-	-	-
*CCDC40*(NM_017950.3):c.1445G>A [tGc/tAc] (p.Cys482Tyr) Missense, rs367601192	0.0000483	0.74673 Figure S2L	0.45678 T	0.38742 T	20.4	0.36828	0.471 D	Blue	Blue	Likely benign	Patient 6
*CCDC40*(NM_017950.3):c.1520A>T [aAg/aTg] (p.Lys507Met) Missense, rs563467821	0.0003944	0.76562 Figure S2M	0.61518 T	0.60299 T	24.3	0.39053	0.855 D	Red	Red	Uncertain significance	-
*CCDC40*(NM_017950.4):c.2440C>T [Cga/Tga] (p.Arg814Ter) Nonsense, rs747233125	0.00000877	0.79007 Figure S2N	-	-	42.0	-	-	-	-	Pathogenic	-
*CCDC65*(NM_033124.4):c.1280A>G [gAt/gGt] (p.Asp427Gly) Missense, rs866658813	0.00000795	0.66012 Figure S2O	0.02439 T	0.42009 T	18.07	0.0939	0.051 N	Green	Green	Likely benign	-
*CCDC114*(NM_144577.3):c.747G>C [ggG/ggC] (p.Gly249=) Synonymous, rs745962113	0.00002048	-	-	-	-	-	-	-	-	Likely benign	-
*CCDC114*(NM_144577.3):c.1032G>A [aaG/aaA] (p.Lys344=) Synonymous, rs753921661	0.00003589	-	-	-	-	-	-	-	-	Likely benign	-
*CEP104*(NM_014704.3):c.2092G>A [Gaa/Aaa] (p.Glu698Lys) Missense, Novel	-	0.79226 Conserved Figure S5M	0.39747 T	0.34116 T	23.3	0.22627	0.421 N	Blue	Blue	-	Pathogenic, Patient 9
*CFAP298*(NM_021254.4):c.77A>C [gAg/gCg] (p.Glu26Ala) Missense, rs138178722	0.002517 *	0.70416 Figure S5N	0.44009 T	0.41632 T	23.7	0.220036	0.042 N	Green	Blue	Benign	-
*DNAAF1*(NM_178452.4):c.241_242delAG, [AGg/g], Frameshift, rs761836563	0.00000398	-	-	-	-	-	-	-	-	Likely pathogenic	-
*DNAAF1*(NM_178452.5):c.1099G>A [Ggg/Agg] (p.Gly367Arg) Missense, rs763129355	0.00008352	0.55445 Figure S3A	0.12497 T	0.13655 T	7.909	0.06188	0.009 N	Green	Green	Likely benign	-
*DNAAF1*(NM_178452.5):c.1205A>T [gAg/gTg] (p.Glu402Val) Missense, rs144034147	0.0001472	0.32593 Figure S3B	0.19559 T	0.15188 T	13.3	0.00175	0.028 N	Green	Green	Likely benign	-
*DNAAF1*(NM_178452.4):c.1698+1G>A, Splice donor, rs139519641	0.0004176	-	-	-	-	-	-	-	-	Likely pathogenic	-
*DNAAF2*(NM_018139.2):c.827C>G [cCg/cGg] (p.Pro276Arg) Missense, rs562712293	0.00001243	0.7484 Figure S3C	0.19839 T	0.11656 T	26.3	0.27849	0.614 D	Blue	Blue	Likely benign	-
*DNAAF3*(NM_001256716):c.53T>G [aTt/aGt] (p.Ile18Ser) Missense, rs537635826	0.0008102	-	0.06425 T	0.40327 T	6.642	0.02051	0.413 N	Green	Green	Likely benign	-
*DNAAF3*(NM_001256714.1):c.1116+5G>C Splice donor, rs1037483400	-	-	-	-	-	-	-	-	-	Uncertain significance	Patient 18; an aberrant effect on splicing is likely.
*DNAAF3*(NM_001256714.1):c.1456C>T, [Cgg/Tgg] (p.Arg486Trp) Missense, rs201929981	0.0002648 *	0.72226 Figure S3D	0.38193 T	0.47481 T	24	0.42485	0.889 D	Red	Blue	Likely benign	-
*DNAAF5*(NM_017802.3):c.788G>A [cGg/cAg] (p.Arg263Gln) Missense, rs201059622	0.0001155 *	0.75769 Figure S3E	0.06998 T	0.42963 T	9.761	0.11179	0.000 N	Green	Green	Likely benign	-
*DNAAF5*(NM_017802.3):c.1131G>T [gtG/gtT] (p.Val377=) Synonymous, rs151119269	0.00001593	0.69489	-	-	-	-	-	-	-	Likely benign	-
*DNAAF5*(NM_017802.4):c.1292T>C [gTc/gCc] (p.Val431Ala) Missense, Novel	-	0.79355 Conserved Figure S3F	0.80849 T	0.83461 D	23.4	0.80713	0.886 D	Red	Red	-	-
*DNAH1*(NM_015512.5):c.4609G>A [Gtg/Atg] (p.Val1537Met) Missense, rs768532151	0.00001204	0.81432 Conserved Figure S1A	0.50078 T	0.54831 T	23.7	0.54201	0.378 N	Blue	Blue	Uncertain significance	-
*DNAH1*(NM_015512.5):c.7238T>C [cTt/cCt] (p.Val2413Ala) Missense, rs1164570685	0.000004013	0.7822 Conserved Figure S1B	0.65577 T	0.72023 T	24.8	0.70589	0.834 D	Red	Red	Uncertain significance	-
*DNAH1*(NM_015512.4):c.11063A>G [tAc/tGc] (p.Tyr3688Cys) Missense, rs369995851	0.0000241	0.85784 Conserved Figure S1C	0.33071 T	0.23649 T	32	0.5602	0.780 D	Red	Blue	Uncertain significance	-
*DNAH5*(NM_001369.2):c.278-3T>C Splice acceptor, rs1244727714	0.00001593	-	-	-	-	-	-	-	-	Uncertain significance	-
*DNAH5*(NM_001369.2):c.2053-23A>C Splice acceptor, rs114717951	0.009576 *	-	-	-	-	-	-	-	-	Benign	-
*DNAH5*(NM_001369.2):c.2253C>A [aaC/aaA] (p.Asn751Lys) Missense, rs115004914	0.009721 *	0.68547 Figure 2B	0.17657 T	0.25336 T	17.21	0.24907	0.000 N	Green	Green	Benign	-
*DNAH5*(NM_001369.2):c.2821G>A [Gtc/Atc] (p.Val941Ile) Missense, rs370080157	0.00009554	0.56602 Figure 2C	0.09101 T	0.40345 T	4.202	0.02609	0.024 N	Green	Green	Likely benign	-
*DNAH5*(NM_001369.2):c.3471G>A [aaG/aaA] (p.Lys1157=) Synonymous, rs865979045	-	-	-	-	-	-	-	-	-	Likely benign	-
*DNAH5*(NM_001369.2):c.4072G>A [Ggc/Agc] (p.Gly1358Ser) Missense, rs752638332	0.00002807	0.75801 Conserved Figure 2D	0.54345 T	0.64213 T	23.2	0.5976	0.734 D	Red	Red	Uncertain significance	-
*DNAH5*(NM_001369.2):c.4331A>G [aAt/aGt] (p.Asn1444Ser) Missense, rs567013299	0.0003152 *	0.7356 Figure 2E	0.35818 T	0.26259 T	6.187	0.25457	0.004 N	Green	Green	Benign	-
DNAH5(NM_001369.2):c.4510G>C [Ggg/Cgg] (p.Gly1504Arg) Missense, rs143567667	0.000728	0.71857 Figure 2F	0.69826 T	0.76696 T	22	0.67108	0.470 D	Red	Red	Uncertain significance	-
*DNAH5*(NM_001369.2):c.4680C>T [ttC/ttT] (p.Phe1560=) Synonymous, rs1283006383	0.000003978	0.76067	-	-	-	-	-	-	-	Likely benign	-
*DNAH5*(NM_001369.2):c.4687G>A [Ggc/Agc] (p.Gly1563Ser) Missense, rs147567352	0.00005569	0.68677 Figure 2G	0.23942 T	0.27354 T	17.56	0.10108	0.003 N	Green	Green	Likely benign	-
*DNAH5*(NM_001369.2):c.4807C>A [Cca/Aca] (p.Pro1603Thr) Missense, rs369137751	0.002391 *	0.84684 Conserved Figure 2H	0.84694 D	0.89805 D	25.8	0.8124	0.897 D	Red	Red	Benign	Pathogenic, Patient 10
*DNAH5*(NM_001369.2):c.5503C>T [Cag/Tag] (p.Gln1835Ter) Nonsense, rs761622153	0.00000799	0.8610 Conserved Figure 2I	-	-	50	-	-	-	-	Pathogenic	Pathogenic, Patient 11
*DNAH5*(NM_001369.2):c.7238T>C [cTt/cCt] (p.Leu2413Pro) Missense, Novel	-	0.78526 Conserved Figure 2J	0.96992 D	0.97908 D	28.6	0.99806	0.842 D	Red	Red	-	-
*DNAH5*(NM_001369.2):c.7619C>T [aCg/aTg] (p.Thr2540Met) Missense, rs144428526	0.00001591	0.74254 Figure 2K	0.08732 T	0.25497 T	12.59	0.0939	0.049 N	Green	Green	Likely benign	-
*DNAH5*(NM_001369.2):c.8765G>A [cGt/cAt] (p.Arg2922His) Missense, rs148539877	0.00009165	0.80492 Conserved v 2L	0.58306 T	0.66715 T	23.5	0.73	0.919 D	Red	Red	Uncertain significance	-
*DNAH5*(NM_001369.2):c.12709G>T [Gtc/Ttc] (p.Val4237Phe) Missense, rs138045391	0.0001117	0.77942 Conserved Figure 2M	0.37894 T	0.34678 T	23	0.56961	0.611 D	Red	Blue	Uncertain significance	-
*DNAH5*(NM_001369.2):c.13492-15T>C, Splice acceptor, rs192514899	0.0003582 *	-	-	-	-	-	-	-	-	Likely benign	-
*DNAH5*(NM_001369.2): Duplication of exons 1-47, Duplication, Novel	-	-	-	-	-	-	-	-	-	-	-
*DNAH6*(NM_001370.1):c.637A>G [Att/Gtt] (p.Ile213Val) Missense, rs774899113	0.0002001	0.82579 Conserved Figure S1D	0.18202 T	0.23812 T	19.52	0.0584	0.000 N	Green	Green	Likely benign	-
*DNAH8*(NM_001206927.1):c.668T>C [aTa/aCa] (p.Ile223Thr) Missense, rs1554195443	-	0.73642 Figure S1E	0.14334 T	0.05175 T	21.8	0.33598	0.324 N	Blue	Blue	Uncertain significance	-
*DNAH8*(NM_001206927.1):c.3061A>G, [Agt/Ggt] (p.Ser1021Gly) Missense, rs865933270	-	0.72356 Figure S1F	0.02018 T	0.44006 T	18.63	0.05153	0.029 N	Green	Green	Likely benign	-
*DNAH8*(NM_001206927.1):c.3289A>G, [Att/Gtt] (p.Ile1097Val) Missense, rs147941001	0.0000963	0.74036 Figure S1G	0.07329 T	0.33784 T	15.18	0.16033	0.001 N	Green	Green	Likely benign	Patient 12
*DNAH8*(NM_001206927.2):c.5789G>A, [cGt/cAt] (p.Arg1930His) Missense, rs758923038	0.00004782	0.74754 Figure S1H	0.05849 T	0.36883 T	16.81	0.04481	0.037 N	Green	Green	Likely benign	Patient 12
*DNAH11*(NM_001277115):c.1241A>G [gAa/gGa] (p.Glu414Gly) Missense, Novel	-	0.77167 Conserved Figure 3B	0.70404 T	0.71692 T	26.5	0.7872	0.896 D	Red	Red	-	-
*DNAH11*(NM_001277115.1):c.2570G>A [cGa/cAa] (p.Arg857Gln) Missense, rs376572966	0.0000444	0.72811 Figure 3C	0.11574 T	0.26196 T	19.89	0.01542	0.519 D	Green	Blue	Likely benign	Patient 13
*DNAH11*(NM_001277115.1):c.2887A>G [Aga/Gga] (p.Arg963Gly) Missense, rs185803317	0.000008103	0.74574 Figure 3D	0.18285 T	0.16981 T	21	0.12243	0.149 N	Green	Green	Likely benign	-
*DNAH11*(NM_001277115.2):c.4202A>G [cAg/cGg] (p.Gln1401Arg) Missense, rs199629774	0.002357 *	0.77106 Figure 3E	0.54477 T	0.48696 T	23.3	0.54486	0.321 N	Blue	Blue	Benign	-
*DNAH11*(NM_001277115.1):c.4775G>T [tGc/tTc] (p.Cys1592Phe) Missense, rs72657327	0.0001981	0.79927 Figure 3F	0.56045 T	0.69537 T	24.1	0.35779	0.639 D	Blue	Red	Uncertain significance	-
*DNAH11*(NM_001277115.1):c.4945-12T>C, Splice acceptor, rs141572016	0.0004917	-	-	-	-	-	-	-	-	Uncertain significance	Patient 14
*DNAH11*(NM_001277115.1):c.5132A>G [cAa/cGa] (p.Gln1711Arg) Missense, rs189432084	0.0005305	0.74319 Figure 3G	0.22617 T	0.17802 T	10.26	0.21439	0.000 N	Green	Green	Likely benign	-
*DNAH11*(NM_001277115.1):c.7729G>A [Gac/Aac] (p.Asp2577Asn) Missense, rs770532527	0.00008837	0.85404 Conserved Figure 3H	0.67362 T	0.71014 T	33	0.81472	0.945 D	Red	Red	Uncertain significance	-
*DNAH11*(NM_001277115.1):c.8023A>G [Att/Gtt] (p.Ile2675Val) Missense, rs72657364	0.001758	0.717 Figure 3I	0.08778 T	0.33151 T	8.696	0.02051	0.000 N	Green	Green	Likely benign	-
*DNAH11*(NM_001277115.2):c.8072A>G [cAg/cGg] (p.Gln2691Arg) Missense, rs183682756	0.0002329	0.72089 Figure 3J	0.21222 T	0.04597 T	21.9	0.1502	0.242 N	Green	Blue	Likely benign	-
*DNAH11*(NM_001277115.1):c.8230C>T [Cgt/Tgt] (p.Arg2744Cys) Missense, rs374826188	0.00008435	0.81402 Conserved Figure 3K	0.64824 T	0.67368 T	27.6	0.71783	0.906 D	Red	Red	Uncertain significance	Patient 15
*DNAH11*(NM_001277115.1):c.8533C>G [Cga/Gga] (p.Arg2845Gly) Missense, rs121908854	0.001171	0.70209 Figure 3L	0.17319 T	0.09501 T	22	0.07246	0.405 N	Green	Blue	Likely benign	-
*DNAH11*(NM_001277115.1):c.9238C>A [Ctg/Atg] (p.Leu3080Met) Missense, Novel	-	0.77741 Conserved Figure 3M	0.67455 T	0.67005 T	24.8	0.48544	0.897 D	Red	Red	-	-
*DNAH11*(NM_001277115.1):c.9305G>A [gGc/gAc] (p.Gly3102Asp) Missense, rs774083447	0.00001615	0.77066 Conserved Figure 3N	0.91256 D	0.91719 D	25.4	0.92601	0.945 D	Red	Red	Uncertain significance	Patient 13
*DNAH11*(NM_001277115.1):c.9380T>G [cTg/cGg] (p.Leu3127Arg) Missense, rs755885697	-	0.76849 Conserved Figure 3O	0.8943 D	0.9065 D	29.3	0.94394	0.945 D	Red	Red	Uncertain significance	-
*DNAH11*(NM_001277115.2):c.10379C>A [aCg/aAg] (p.Thr3460Lys) Missense, rs573384750	0.0001832	0.81088 Conserved Figure 3P	0.66914 T	0.69202 T	24.7	0.61646	0.625 D	Red	Red	Likely benign	-
*DNAH11*(NM_001277115.1):c.11267G>A [cGc/cAc] (p.Arg3756His) Missense, rs554657293	0.00006853	0.73608 Figure 3Q	0.88418 D	0.89161 D	21.6	0.7872	0.388 N	Red	Red	Uncertain significance	-
*DNAH11*(NM_001277115.1):c.11839+1G>A Splice donor, Novel	-	-	-	-	-	-	-	-	-	-	Pathogenic, Patient 15
*DNAH11*(NM_001277115.1):c.12344T>G [aTt/aGt] (p.Ile4115Ser) Missense, rs371418299	0.0005882 *	0.80147 Figure 3R	0.24391 T	0.00625 T	23.6	0.58263	0.529 D	Blue	Blue	Likely benign	-
*DNAH11*(NM_001277115.1):c.13010G>A [aGc/aAc] (p.Ser4337Asn) Missense, rs759646661	0.00002809	0.7162 Figure 3S	0.04369 T	0.32012 T	22	0.05495	0.059 N	Green	Green	Likely benign	Patient 13
*DNAH11*(NM_001277115.1):c.13120G>A [Gtg/Atg] (p.Val4374Met) Missense, rs560018723	-	0.81781 Figure 3T	0.35791 T	0.02566 T	24.6	0.63525	0.688 D	Red	Blue	Uncertain significance	Patient 14
*DNAI1*(NM_012144.3):c.40C>T [Cat/Tat] (p.His14Tyr) Missense, rs146501326	0.0001551	0.49721 Figure S4A	0.58033 T	0.52983 T	17.25	0.34483	0.056 N	Green	Green	Likely benign	-
*DNAI1*(NM_012144.3):c.47A>G [cAg/cGg] (p.Gln16Arg) Missense, rs148701985	0.0003422	0.68698 Figure S4B	0.49514 T	0.5142 T	21.5	0.19284	0.007 N	Green	Green	Likely benign	-
*DNAI1*(NM_012144.4):c.81+20T>C Splice donor, rs572257884	0.000249	-	-	-	-	-	-	-	-	Uncertain significance	-
*DNAI1*(NM_012144.4):c.274_281delAAGCCTAT (p.Lys92Trpfs) Frameshift, Novel	-	-	-	-	-	-	-	-	-	-	-
*DNAI1*(NM_012144.3):c.1173C>G [atC/atG] (p.Ile391Met) Missense, rs151097256	0.00009949	0.7792 Figure S4C	0.52782 T	0.34281 T	21	0.5304	0.042 N	Blue	Blue	Likely benign	-
*DNAI1*(NM_012144.3):c.1265_1267del [tTCTgc/tgc] (p.Phe422del) Inframe deletion, rs567346433	0.0006057	-	-	-	-	-	-	-	-	Likely benign	-
*DNAI2*(NM_023036.4):c.685T>G [Tcc/Gcc] (p.Ser229Ala) Missense, rs576683556	0.0002585 *	0.83226 Conserved Figure S4D	0.213 T	0.18233 T	22.4	0.10108	0.059 N	Green	Green	Likely benign	-
*DNAI2*(NM_023036.4):c.891G>A [atG/atA] (p.Met297Ile) Missense, rs750750518	0.00003181	0.75677 Figure S4E	0.09009	0.13899 T	15.3	0.10108	0.272 N	Green	Green	Likely benign	-
*DNAI2*(NM_023036.4):c.1318G>C [Gag/Cag] (p.Glu440Gln) Missense, rs182986650	0.00002387	0.74919 Figure S4F	0.12935 T	0.21396 T	22.3	0.24349	0.037 N	Green	Green	Likely benign	-
*DNAI2*(NM_023036.4):c.1574C>T [gCg/gTg] (p.Ala525Val) Missense, rs145602856	0.0007551	0.61318 Figure S4G	0.47021 T	0.41194 T	10.79	0.26002	0.323 N	Blue	Green	Likely benign	-
*DNAI2*(NM_023036.4):c.1715C>T [cCa/cTa] (p.Pro572Leu) Missense, rs151241589	0.001541 *	0.46808 Figure S4H	0.53676 T	0.18762 T	12.81	0.18967	0.059 N	Green	Green	Likely benign	-
*DRC1*(NM_145038.4):c.1090G>A [Gag/Aag] (p.Glu364Lys) Missense, rs184506507	0.00000398	0.73269 Figure S5A	0.31284 T	0.10997 T	27.8	0.47558	0.743 D	Red	Blue	Likely benign	Likely pathogenic, Patient 16
*DRC1*(NM_145038.4):c.1146G>C [gaG/gaC] (p.Glu382Asp) Missense, Novel	-	0.76693 Figure S5B	0.04985 T	0.32928 T	17.61	0.06188	0.022 N	Green	Green	-	-
*DRC1*(NM_145038.5):c.2081G>C [aGg/aCg] (p.Arg694Thr) Missense, rs372797665	0.0001233	0.77074 Conserved Figure S5C	0.85587 D	0.90334 D	35	0.82668	0.935 D	Red	Red	Uncertain significance	Likely pathogenic, Patient 16
*HYDIN*(NM_001270974.1):c.1003G>T [Gta/Tta] (p.Val335Leu) Missense, rs755584531	0.000076450	0.63033 Figure S5D	0.07565 T	0.43605 T	10.91	0.25457	0.009 N	Green	Green	Likely benign	Patients 17-18
*HYDIN*(NM_001270974.1):c.9638C>G [cCc/cGc] (p.Pro3213Arg) Missense, Novel	-	0.60436 Figure S5E	0.0133 T	0.46351 T	22.5	0.32697	0.530 D	Blue	Blue	-	Likely pathogenic, Patients 17-18
*HYDIN*(NM_001270974.2):c.11173C>T [Cgg/Tgg] (p.Arg3725Trp) Missense, rs79417681	0.00008865	0.6387 Figure S5F	0.00646 T	0.43195 T	20.6	0.22036	0.506 D	Blue	Blue	Likely benign	-
*NME8*(NM_016616.4):c.1630G>A [Gca/Aca] (p.Ala544Thr) Missense, rs140494494	0.0005613 *	0.68484 Figure S5O	0.78209 T	0.82492 D	24.7	0.76421	0.945 D	Red	Red	Likely benign	-
*NME8*(NM_016616.4):c.271-27C>T Splice acceptor, rs117149381	0.01787 *	-	-	-	-	-	-	-	-	Benign	-
*OFD1*(NM_003611.3):c.2927A>C [aAg/aCg ](p.Lys976Thr) Missense, rs1458317780	0.000005470	0.68731 Figure S5R	0.97362 D	0.97841 D	24.2	0.71627	0.841 D	Red	Red	Uncertain significance	See Results
*RSPH1*(NM_080860.4):c.730G>A [Gca/Aca] (p.Ala244Thr) Missense, rs150400022	0.0007761 *	0.60579 Figure S5G	0.15074 T	0.19078 T	0.478	0.02609	0.009 N	Green	Green	Likely benign	-
*RSPH4A*(NM_001010892.3):c.650A>C [tAc/tCc] (p.Tyr217Ser) Missense, rs762313827	0.00006861	0.83409 Conserved Figure S5H	0.36055 T	0.3837 T	26.6	0.68023	0.919 D	Red	Blue	Uncertain significance	-
*RSPH4A*(NM_001010892):c.1410C>G [atC/atG] (p.Ile470Met) Missense, rs775326896	0.000027850	0.7634 Figure S5I	0.25237 T	0.11706 T	19.44	0.5519	0.781 D	Red	Blue	Likely benign	-
*RSPH9*(NM_001193341.1):c.365G>A [gGt/gAt] (p.Gly122Asp) Missense, rs1195999841	0.00000398	0.66683 Figure S5J	0.41053 T	0.29917 T	21	0.35566	0.423 N	Blue	Blue	Uncertain significance	-
*SPAG1*(NM_172218.2):c.957T>A [gtT/gtA] (p.Val319=) Synonymous, rs146528350	0.0008908 *	-	-	-	-	-	-	-	-	Likely benign	-
*SPAG1*(NM_172218.2):c.1435+16C>T Splice donor, rs148767962	0.0002789	-	-	-	-	-	-	-	-	Likely benign	-
*ZMYND10*(NM_015896.4):c.1019G>A [cGg/cAg] (p.Arg340Gln) Missense, rs148328402	0.002811 *	0.66864 Figure S5P	0.31943 T	0.30892 T	18.76	0.14679	0.010 N	Green	Green	Benign	-

**Table 3:** Patients with double/triple heterozygous or homozygous variants. Predictions of pathogenicity of the variants are in Table 2. Heterozygous variants in these patients are not shown. XLR, X-linked recessive; Homo, homozygous. Novel variants are bolded. ^§^ This intronic change generates a cryptic donor splice site, which inserts an intronic sequence between exons 9 and 10^8^. The term ‘triple heterozygous’ signifies the presence of three variants in the same gene, as in Patients 13 and 14. As phasing was done, it is unclear on which allele the triple heterozygous variants resided, as pathogenic compound heterozygous variants could result in autosomal recessive disease.

Patient	First	Second	Third	Clinical Assessment
1 Homo	*C1orf127*:c.337C>T (p.Arg113Ter)	*C1orf127*:c.337C>T (p.Arg113Ter)	-	Heterotaxy, asplenia, midline liver, pulmonary stenosis, interrupted inferior vena cava, bilateral superior vena cava, and right aortic arch.
2 Homo	*C1orf127*:c.337C> (p.Arg113Ter)	*C1orf127*:c.337C> (p.Arg113Ter)	-	Dextrocardia, pulmonary stenosis, respiratory infections. Parents are heterozygous for *EP400* and asymptomatic. Family members with congenital heart anomalies.
	*EP400*:c.323C>T (p.Ala108Val) rs762116055-Likely benign	*EP400*:c.323C>T (p.Ala108Val) rs762116055 - Likely benign	-	
3	*CCDC39*:c.1073C>T (p.Thr358Ile)	*CCDC39*:c.1076A>C (p.Lys359Thr)	-	Prematurity (32 weeks’ gestation) with persistent atelectasis. Parental studies revealed the variants are in *cis* phase.
4 Homo	*CCDC39*:c.1167+1261A>G	*CCDC39*:c.1167+1261A>G	-	Two cousins with homozygosity and diagnostic features of PCD. ^§^
5	*CCDC39*:c.1363-3delC	*CCDC39*:c.1781C>T (p.Thr594Ile)	-	Clinical features of PCD. ^§^
6	*CCDC40*:c.1445G>A (p.Cys482Tyr)	*CCDC40*:c.257A>G (p.Tyr86Cys)	-	Heterotaxy syndrome (isomerism).
7	*CCDC40*:c.850G>C (p.Asp284His)	*CCDC40*:c.697G>A (p.Asp233Asn)	-	Recurrent sinusitis.
8 Homo	*CCDC40*:c.62G>T (p.Gly21Val)	*CCDC40*:c.62G>T (p.Gly21Val)	-	Two siblings with sinopulmonary infections (including chronic otorrhea) from early infancy with ultrastructural defects in the cilia (significant microtubular disorganizations, including distorted dynein arms and absent inner dynein arms).
9 Homo	*CEP104*:c.2092G>A (p.Glu698Lys)	*CEP104*:c.2092G>A (p.Glu698Lys)	-	Joubert syndrome 25 (MIM#616781). Parents are asymptomatic carriers.
10 Homo	*DNAH5*:c.4807C>A (p.Pro1603Tyr)	*DNAH5*:c.4807C>A (p.Pro1603Tyr)	-	Two sisters with bronchiectasis and chronic sinusitis. Parents are heterozygous and asymptomatic. Mother had recurrent abortions and an ectopic pregnancy.
11 Homo	*DNAH5*:c.5503C>T (p.Gln1835Ter)	*DNAH5*:c.5503C>T (p.Gln1835Ter)	-	Two sisters with chronic respiratory infections and ultrastructural defects in the cilia (significant microtubular disorganizations, including distorted outer dynein arms).
12	*DNAH8*:c.3289A>G (p.Ile1097Val)	*DNAH8*:c.5789G>A (p.Arg1930His)	-	Clinical features of PCD.
13	*DNAH11*:c.2570G>A (p.Arg857Gln)	*DNAH11*:c.9305G>A (p.Gly3102Asp)	*DNAH11*:c.13010G>A (p.Ser4337Asn)	Recurrent respiratory infections from childhood and non-motile sperms. Since parents were not tested, phasing of variants could not be performed.
14	*DNAH11*:c.13120G>A (p.Val4374Met)	*DNAH11*:c.4945-12T>C	*DNAH11*:c.5132A>G (p.Gln1711Arg)	Chronic sinusitis. Since parents were not tested, phasing of variants could not be performed.
15	*DNAH11*:c.8230C>T (p.Arg2744Cys)	*DNAH11*:c.11839+1G>A	-	Situs inversus totalis with dextrocardia.
16	*DRC1*:c.1090G>A (p.Glu364Lys)	*DRC1*:c.2081G>C (p.Arg694Thr)	-	Clinical features of PCD. Parents are first cousins. The mother also has clinical features of PCD with the same two variants. The father is not tested.
17	*HYDIN*:c.1003G>T (p.Val335Leu)	*HYDIN*:c.9638C>G (p.Pro3213Arg)	-	Clinical features of PCD.
18	*HYDIN*:c.1003G>T (p.Val335Leu)	*HYDIN*:c.9638C>G (p.Pro3213Arg)	-	Prematurity (33 weeks’ gestation) with clinical features of PCD.
	*DNAAF3*:c.1053+5G>C	*DNAAF3*:c.1116+5G>C	-	

## Supplementary Materials

The following are available online at https://www.mdpi.com/article/10.3390/jcm10215102/s1, Figure S1: Multiple sequence alignment of dynein axonemal heavy chain proteins (other than DNAH5 and DNAH11); Figure S2: Multiple sequence alignment of coiled-coil domain-containing (CCDC) proteins; Figure S3: Multiple sequence alignment of dynein axonemal assembly factor (DNAAF) proteins; Figure S4: Multiple sequence alignment of dynein axonemal intermediate chain (DNAI) proteins; Figure S5: Multiple sequence alignment of other proteins; Table S1: PCD genes covered by the Centogene^®^ comprehensive pulmonary disease panel; Table S2: Details of genetic tests done; Table S3: NCBI RefSeq accession of protein sequences used for multiple sequence alignment.

## References

[1] Butterfield R. (2017). Primary Ciliary Dyskinesia. Pediatr. Rev..

[2] Knowles M.R., Zariwala M., Leigh M. (2016). Primary Ciliary Dyskinesia. Clin. Chest Med..

[3] Hosie P.H., Fitzgerald D.A., Jaffe A., Birman C.S., Rutland J., Morgan L.C. (2014). Presentation of primary ciliary dyskinesia in children: 30 years’ experience. J. Paediatr. Child Health.

[4] O’Connor M.G., Griffiths A., Iyer N.P., Shapiro A., Wilson K.C., Thomson C.C. (2019). Summary for Clinicians: Diagnosis of Primary Ciliary Dyskinesia. Ann. Am. Thorac. Soc..

[5] Lucas J.S., Davis S.D., Omran H., Shoemark A. (2020). Primary ciliary dyskinesia in the genomics age. Lancet Respir. Med..

[6] Horani A., Ferkol T.W. (2018). Advances in the Genetics of Primary Ciliary Dyskinesia: Clinical Implications. Chest.

[7] Horani A., Ferkol T.W., Dutcher S.K., Brody S.L. (2016). Genetics and biology of primary ciliary dyskinesia. Paediatr. Respir. Rev..

[8] Merveille A.-C., Davis E.E., Becker-Heck A., Legendre M., Amirav I., Bataille G., Belmont J.W., Beydon N., Billen F., Clément A. (2010). CCDC39 is required for assembly of inner dynein arms and the dynein regulatory complex and for normal ciliary motility in humans and dogs. Nat. Genet..

[9] Oda T., Yanagisawa H., Kamiya R., Kikkawa M. (2014). A molecular ruler determines the repeat length in eukaryotic cilia and flagella. Science.

[10] Zariwala M.A., Knowles M.R., Leigh M.W., Adam M.P., Ar-dinger H.H., Pagon R.A., Wallace S.E., Bean L.J., Mirzaa G., Amemiya A. (1993). Primary Ciliary Dyskinesia. GeneReviews®.

[11] Antony D., Becker-Heck A., Zariwala M.A., Schmidts M., Onoufriadis A., Forouhan M., Wilson R., Taylor-Cox T., Dewar A., Jackson C. (2013). Mutations inCCDC39andCCDC40are the Major Cause of Primary Ciliary Dyskinesia with Axonemal Disorganization and Absent Inner Dynein Arms. Hum. Mutat..

[12] Antony D., Brunner H.G., Schmidts M. (2021). Ciliary Dyneins and Dynein Related Ciliopathies. Cells.

[13] Baz-Redón N., Rovira-Amigo S., Fernández-Cancio M., Castillo-Corullón S., Cols M., Caballero-Rabasco M.A., Asensio Ó., de Vicente C.M., Martínez-Colls M.D.M., Torrent-Vernetta A. (2020). Immunofluorescence Analysis as a Diagnostic Tool in a Spanish Cohort of Patients with Suspected Primary Ciliary Dyskinesia. J. Clin. Med..

[14] O’Connor M.G., Horani A., Shapiro A.J. (2021). Progress in Diagnosing Primary Ciliary Dyskinesia: The North American Perspective. Diagnostics.

[15] Werner C., Onnebrink J.G., Omran H. (2015). Diagnosis and management of primary ciliary dyskinesia. Cilia.

[16] Shoemark A., Frost E., Dixon M., Ollosson S., Kilpin K., Patel M., Scully J., Rogers A.V., Mitchison H.M., Bush A. (2017). Accuracy of Immunofluorescence in the Diagnosis of Primary Ciliary Dyskinesia. Am. J. Respir. Crit. Care Med..

[17] Shapiro A.J., Dell S.D., Gaston B., O’Connor M., Marozkina N., Manion M., Hazucha M.J., Leigh M.W. (2020). Nasal Nitric Oxide Measurement in Primary Ciliary Dyskinesia. A Technical Paper on Standardized Testing Protocols. Ann. Am. Thorac. Soc..

[18] Knowles M.R., Ostrowski L.E., Leigh M.W., Sears P.R., Davis S.D., Wolf W.E., Hazucha M.J., Carson J.L., Olivier K.N., Sagel S.D. (2014). Mutations inRSPH1Cause Primary Ciliary Dyskinesia with a Unique Clinical and Ciliary Phenotype. Am. J. Respir. Crit. Care Med..

[19] Horani A., Ferkol T.W. (2021). Understanding Primary Ciliary Dyskinesia and Other Ciliopathies. J. Pediatr..

[20] Shapiro A.J., Zariwala M.A., Ferkol T.W., Davis S.D., Sagel S.D., Dell S.D., Rosenfeld M., Olivier K.N., Milla C., Daniel S.J. (2016). Diagnosis, monitoring, and treatment of primary ciliary dyskinesia: PCD foundation consensus recommendations based on state of the art review. Pediatr. Pulmonol..

[21] AlSaadi M.M., Gaunt T.R., Boustred C.R., Guthrie P.A.I., Liu X., Lenzi L., Rainbow L., Hall N., Alharbi K.K., Day I.N.M. (2012). From a Single Whole Exome Read to Notions of Clinical Screening: Primary Ciliary Dyskinesia and RSPH9 p.Lys268del in the Arabian Peninsula. Ann. Hum. Genet..

[22] United Arab Emirates Population (2021)—Worldometer. https://www.worldometers.info/world-population/united-arab-emirates-population/.

[23] Alsamri M.T., Alabdouli A., Alkalbani A.M., Iram D., Antony P., Vijayan R., Souid A.-K. (2020). Genetic variants in children with chronic respiratory diseases. Pediatr. Pulmonol..

[24] Shamseldin H.E., Al Mogarri I., Alqwaiee M.M., Alharbi A.S., Baqais K., AlSaadi M., AlAnzi T., Alhashem A., Saghier A., Ameen W. (2020). An exome-first approach to aid in the diagnosis of primary ciliary dyskinesia. Qual. Life Res..

[25] Sivadas A., Scaria V. (2018). Pharmacogenomic survey of Qatari populations using whole-genome and exome sequences. Pharm. J..

[26] Hammoudeh S., Gadelhak W., Janahi I.A. (2018). Primary ciliary dyskinesia among Arabs: Where do we go from here?. Paediatr. Respir. Rev..

[27] Reish O., Slatkin M., Chapman-Shimshoni D., Elizur A., Chioza B., Castleman V., Mitchison H.M. (2010). Founder Mutation(s) in theRSPH9Gene Leading to Primary Ciliary Dyskinesia in Two Inbred Bedouin Families. Ann. Hum. Genet..

[28] Stannard W., Rutman A., Wallis C., O’Callaghan C. (2004). Central Microtubular Agenesis Causing Primary Ciliary Dyskinesia. Am. J. Respir. Crit. Care Med..

[29] Castleman V.H., Romio L., Chodhari R., Hirst R.A., de Castro S.C.P., Parker K.A., Ybot-Gonzalez P., Emes R.D., Wilson S.W., Wallis C. (2009). Mutations in Radial Spoke Head Protein Genes RSPH9 and RSPH4A Cause Primary Ciliary Dyskinesia with Central-Microtubular-Pair Abnormalities. Am. J. Hum. Genet..

[30] McLaren W., Gil L., Hunt S.E., Riat H.S., Ritchie G.R.S., Thormann A., Flicek P., Cunningham F. (2016). The Ensembl Variant Effect Predictor. Genome Biol..

[31] Edgar R.C. (2004). MUSCLE: Multiple sequence alignment with high accuracy and high throughput. Nucleic Acids Res..

[32] Capra J.A., Singh M. (2007). Predicting functionally important residues from sequence conservation. Bioinformatics.

[33] Woods C.G., Cox J., Springell K., Hampshire D.J., Mohamed M.D., McKibbin M., Stern R., Raymond F.L., Sandford R., Sharif S.M. (2006). Quantification of Homozygosity in Consanguineous Individuals with Autosomal Recessive Disease. Am. J. Hum. Genet..

[34] Gunning A.C., Fryer V., Fasham J., Crosby A.H., Ellard S., Baple E.L., Wright C.F. (2021). Assessing performance of pathogenicity predictors using clinically relevant variant datasets. J. Med. Genet..

[35] Ba W., Yan Y., Reijnders M.R.F., Schuurs-Hoeijmakers J.H.M., Feenstra I., Bongers E.M.H.F., Bosch D.G.M., de Leeuw N., Pfundt R., Gilissen C. (2016). TRIOloss of function is associated with mild intellectual disability and affects dendritic branching and synapse function. Hum. Mol. Genet..

[36] Citti A., Peca D., Petrini S., Cutrera R., Biban P., Haass C., Boldrini R., Danhaive O. (2013). Ultrastructural Characterization of Genetic Diffuse Lung Diseases in Infants and Children: A Cohort Study and Review. Ultrastruct. Pathol..

[37] Kurkowiak M., Zietkiewicz E., Witt M. (2015). Recent advances in primary ciliary dyskinesia genetics. J. Med. Genet..

