# Supplementary material for: A Study on the Genetics of Primary Ciliary Dyskinesia
Source: J Clin Med. 2021 Oct 30;10(21):5102. doi: 10.3390/jcm10215102 (PMC8584573; doi:10.3390/jcm10215102)
Supplement: Supplementary file 1 [file jcm-10-05102-s001.zip › jcm-1436043-supplementary.pdf]

A Study on the Genetics of Primary Ciliary Dyskinesia

Mohammed T. Alsamri[1], Amnah Alabdouli[1], Durdana Iram[1], Alia M. Alkalbani[1],

Ayesha S. Almarzooqi[2], Abdul-Kader Souid[2,*], Ranjit Vijayan[3,4,*]

[1] Department of Pediatrics, Tawam Hospital, Al Ain, United Arab Emirates

[2] Department of Pediatrics, College of Medicine and Health Sciences, United Arab Emirates University, Al Ain, United Arab Emirates

[3] Department of Biology, College of Science, United Arab Emirates University, Al Ain, United Arab Emirates

[4] Big Data Analytics Center, United Arab Emirates University, Al Ain, United Arab Emirates

*Correspondences*: Abdul-Kader Souid (asouid@uaeu.ac.ae) and Ranjit Vijayan (ranjit.v@uaeu.ac.ae)

**SUPPLEMENTARY MATERIALS**

**Table S1.** PCD genes covered by the Centogene® comprehensive pulmonary disease panel (Source: https://www.centogene.com/science/centopedia/ comprehensive-pulmonary-disease-panel.html; accessed 17 September 2021)

| Gene | OMIM (Gene) | Associated diseases (OMIM) |
|---|---|---|
| *CCDC39* | 613798 | Ciliary dyskinesia, primary, 14 |
| *CCDC40* | 613799 | Ciliary dyskinesia, primary, 15 |
| *DNAAF1* | 613190 | Ciliary dyskinesia, primary, 13 |
| *DNAAF2* | 612517 | Ciliary dyskinesia, primary, 10 |
| *DNAH11* | 603339 | Primary ciliary dyskinesia type 7, with or without situs inversus |
| *DNAH5* | 603335 | Primary ciliary dyskinesia type 3, with or without situs inversus |
| *DNAH9* | 603330 | |
| *DNAI1* | 604366 | Primary ciliary dyskinesia type 1, with or without situs inversus |
| *DNAI2* | 605483 | Primary ciliary dyskinesia type 9, with or without situs inversus |
| *DNAL1* | 610062 | Ciliary dyskinesia, primary, 16 |
| *NME8* | 607421 | Ciliary dyskinesia, primary, 6 |
| *RSPH1* | 609314 | Primary ciliary dyskinesia, 24 |
| *RSPH4A* | 612647 | Ciliary dyskinesia, primary, 11 |
| *RSPH9* | 612648 | Ciliary dyskinesia, primary, 12 |

**Table S2.** Details of genetic tests done

| Patient | Comprehensive pulmonary disease panel | Whole (diagnostic) exome sequencing | Chromosomal Microarray | Targeted deletion/ duplication analysis | Single gene sequencing |
|---|---|---|---|---|---|
| 1 | Test done, variant in PCD-related gene detected | Test not done | Test not done | Test done for DNAH1; negative result | Test not done |
| 2 | Test not done | Test done, variant in PCD-related gene detected | Test done, abnormalities in PCD-related genes not found | Test not done | Test not done |
| 3 | Test not done | Test done, variant in PCD-related gene detected | Test not done | Test not done | Test not done |
| 4 | Test done, variant in PCD-related gene detected | Test not done | Test not done | Test not done | Test not done |
| 5 | Test done, variant in PCD-related gene detected | Test not done | Test not done | Test not done | Test not done |
| 6 | Test done, variant in PCD-related gene detected | Test not done | Test not done | Test not done | Test not done |
| 7 | Test done, variant in PCD-related gene detected | Test not done | Test not done | Test not done | Test not done |
| 8 | Test done, variant in PCD-related gene detected | Test not done | Test not done | Test not done | Test not done |
| 9 | Test done, variant in PCD-related gene detected | Test done, variant in PCD-related gene detected | Test not done | Test not done | Test not done |
| 10 | Test not done | Test not done | Test not done | Test not done | Test done, variant in *DNAH5* detected |
| 11 | Test done, variant in PCD-related gene detected | Test not done | Test done, duplication of exons 1-47 of *DNAH5* found | Test done for *DNAH5*; negative result | Test not done |
| 12 | Test done, variant in PCD-related gene detected | Test not done | | Test not done | Test not done |
| 13 | Test done, variant in PCD-related gene detected | Test not done | Test not done | Test not done | Test not done |
| 14 | Test done, variant in PCD-related gene detected | Test not done | Test done, abnormalities in PCD-related genes not found | Test not done | Test not done |
| 15 | Test not done | Test done, variant in PCD-related gene detected | Test done, abnormalities in PCD-related genes not found | Test not done | Test not done |

| | | | | | |
|---|---|---|---|---|---|
| 16 | Test not done | Test done, variant in PCD-related gene detected | Test not done | Test not done | Test done, two variants in *DRC1* detected |
| 17 | Test done, variant in PCD-related gene detected | Test not done | Test not done | Test not done | Test not done |
| 18 | Test done, variant in PCD-related gene detected | Test not done | Test not done | Test done for DNAH5; negative result | Test not done |
| 19 | Test done, variant in PCD-related gene detected | Test done, variant in PCD-related gene detected | Test not done | Test not done | Test not done |
| 20 | Test done, variant in PCD-related gene detected | Test not done | Test not done | Test not done | Test not done |
| 21 | Test done, variant in PCD-related gene detected | Test not done | Test not done | Test not done | Test not done |
| 22 | Test not done | Test not done | Test not done | Test not done | Test done, variant in *CCDC39* and variant in *DNAH5* detected |
| 23 | Test done, variant in PCD-related gene detected | Test not done | Test not done | Test not done | Test not done |
| 24 | Test done, variant in PCD-related gene detected | Test not done | Test not done | Test not done | Test not done |
| 25 | Test done, variant in PCD-related gene detected | Test not done | Test done, abnormalities in PCD-related genes not found | Test not done | Test not done |
| 26 | Test done, variant in PCD-related gene detected | Test done, variant in PCD-related gene detected | Test not done | Test not done | Test not done |
| 27 | Test done, variant in PCD-related gene detected | Test not done | Test not done | Test not done | Test not done |
| 28 | Test done, variant in PCD-related gene detected | Test not done | Test not done | Test not done | Test not done |
| 29 | Test done, variant in PCD-related gene detected | Test not done | Test not done | Test not done | Test not done |
| 30 | Test not done | Test not done | Test not done | Test not done | Test done, variant in *DNAH5* detected |
| 31 | Test not done | Test not done | Test not done | Test not done | Test done, variant in *DNAH5* detected |

| | | | | | |
|---|---|---|---|---|---|
| 32 | Test not done | Test not done | Test not done | Test not done | Test done, variant in *DNAH5* detected |
| 33 | Test not done | Test done, variant in PCD-related gene detected | Test not done | Test not done | Test not done |
| 34 | Test done, variant in PCD-related gene detected | Test not done | Test not done | Test not done | Test not done |
| 35 | Test done, variant in PCD-related gene detected | Test not done | Test not done | Test not done | Test not done |
| 36 | Test done, variant in PCD-related gene detected | Test not done | Test not done | Test done for DNAI1; negative result | Test not done |
| 37 | Test not done | Test done, variant in PCD-related gene detected | Test done, abnormalities in PCD-related genes not found | Test not done | Test not done |
| 38 | Test not done | Test not done | Test not done | Test not done | Test done, variant in *DNAH11* detected |
| 39 | Test done, variant in PCD-related gene detected | Test not done | Test not done | Test not done | Test not done |
| 40 | Test not done | Test done, variant in PCD-related gene detected | Test done, abnormalities in PCD-related genes not found | Test not done | Test not done |
| 41 | Test done, variant in PCD-related gene detected | Test not done | Test not done | Test not done | Test not done |
| 42 | Test not done | Test done, variant in PCD-related gene detected | Test done, abnormalities in PCD-related genes not found | Test not done | Test not done |
| 43 | Test done, variant in PCD-related gene detected | Test not done | Test not done | Test not done | Test not done |
| 44 | Test done, variant in PCD-related gene detected | Test not done | Test not done | Test not done | Test not done |
| 45 | Test done, variant in PCD-related gene detected | Test not done | Test done, duplication of exons 1-47 of DNAH5 found | Test not done | Test not done |
| 46 | Test not done | Test done, variant in PCD-related gene detected | Test done, abnormalities in PCD-related genes not found | Test not done | Test not done |

| | | | | | |
|---|---|---|---|---|---|
| 47 | Test done, variant in PCD-related gene detected | Test done, variant in PCD-related gene *not* detected | Test done, abnormalities in PCD-related genes not found | Test not done | Test not done |
| 48 | Test done, variant in PCD-related gene detected | Test done, variant in PCD-related gene *not* detected | Test not done | Test not done | Test not done |
| 49 | Test done, variant in PCD-related gene detected | Test not done | Test not done | Test not done | Test not done |
| 50 | Test not done | Test done, varaints in PCD-related genes *not* detected | Test done, duplication of exons 1-47 of DNAH5 found | Test not done | Test not done |
| 51 | Test done, variant in PCD-related gene detected | Test not done | Test not done | Test not done | Test not done |
| 52 | Test done, variant in PCD-related gene detected | Test not done | Test not done | Test not done | Test not done |
| 53 | Test done, variant in PCD-related gene detected | Test not done | Test not done | Test not done | Test not done |
| 54 | Test done, variant in PCD-related gene detected | Test not done | Test not done | Test not done | Test not done |
| 55 | Test done, variant in PCD-related gene detected | Test not done | Test not done | Test not done | Test not done |
| 56 | Test done, variant in PCD-related gene detected | Test not done | Test not done | Test done for DNAH5; negative result | Test not done |
| 57 | Test done, variant in PCD-related gene detected | Test not done | Test not done | Test not done | Test not done |
| 58 | Test done, variant in PCD-related gene detected | Test done, variant in PCD-related gene detected | Test not done | Test not done | Test not done |
| 59 | Test done, variant in PCD-related gene detected | Test not done | Test not done | Test not done | Test not done |
| 60 | Test done, variant in PCD-related gene detected | Test not done | Test not done | Test not done | Test not done |
| 61 | Test done, variant in PCD-related gene detected | Test not done | Test not done | Test not done | Test not done |
| 62 | Test not done | Test not done | Test done, duplication of exons 1-47 of DNAH5 found | Test not done | Test done, variant in *DNAH5* detected |
| 63 | Test not done | Test not done | Test done, duplication of exons 1-47 of DNAH5 found | Test not done | Test not done |

| | | | | | |
|---|---|---|---|---|---|
| 64 | Test done, variant in PCD-related gene detected | Test not done | | Test not done | Test not done |
| 65 | Test done, variant in PCD-related gene detected | Test not done | Test done, abnormalities in PCD-related genes not found | Test not done | Test not done |
| 66 | Test done, variant in PCD-related gene detected | Test not done | Test not done | Test not done | Test not done |

**Table S3**. NCBI RefSeq accession of protein sequences used for multiple sequence alignment

| Protein | Human | Chimpanzee | Mouse | Rat | Dog | Horse | Bovine | Frog | Chicken | Zebrafish |
|---|---|---|---|---|---|---|---|---|---|---|
| ARMC4 | NP_060546.2 | XP_016818326.2 | XP_030106482.1 | XP_008770032.1 | XP_535146.3 | XP_023488011.1 | XP_024856406.1 | XP_017950189.1 | XP_003640684.3 | XP_005156286.1 |
| C1orf127 | NP_001164225.1 | XP_016809392.1 | NP_001355764.1 | XP_017449424.1 | - | XP_023484158.1 | - | XP_017951136.1 | - | XP_021325672.1 |
| CCDC39 | NP_852091.1 | XP_016797828.1 | NP_080498.1 | NP_001101137.1 | XP_545213.3 | XP_023479118.1 | NP_001192985.1 | NP_001072342.1 | XP_422777.4 | XP_001344625.2 |
| CCDC40 | NP_060420.2 | XP_009431725.2 | NP_001351696.1 | NP_001128160.1 | XP_005624081.1 | XP_023507634.1 | XP_010814878.1 | XP_002937644.2 | XP_024997485.1 | NP_001258742.1 |
| CCDC65 | NP_149115.2 | NP_001233475.1 | NP_705738.1 | NP_001014225.1 | XP_534816.1 | XP_001504180.1 | NP_001033255.1 | XP_012813228.3 | XP_025001449.1 | XP_005165756.1 |
| CEP104 | NP_055519.1 | XP_016808323.1 | NP_808341.1 | NP_659550.2 | XP_022274677.1 | XP_023491684.1 | XP_015330669.1 | XP_012822790.2 | XP_004947417.1 | XP_005167010.1 |
| CFAP298 | NP_067077.1 | NP_001337257.1 | NP_080778.2 | NP_001008289.1 | XP_848523.1 | XP_001498633.1 | NP_001039872.1 | NP_001086851.1 | NP_001006258.1 | NP_956382.1 |
| DNAAF1 | NP_848547.4 | XP_016785762.2 | NP_080924.1 | NP_001014176.1 | XP_536764.3 | XP_023493171 .1 | NP_001030422.2 | XP_004913593.2 | - | NP_001306060.1 |
| DNAAF2 | NP_060609.2 | XP_509930.3 | NP_081545.3 | NP_001014219.3 | XP_005623972.1 | XP_023480449.1 | NP_001069492.2 | NP_001072474.1 | XP_421456.5 | NP_001028272.1 |
| DNAAF3 | NP_001243643.1 | XP_016789630.1 | NP_001028720.1 | NP_001258044.1 | XP_005616232.1 | XP_014586276.1 | XP_005219742.3 | XP_012822876.2 | | XP_021326065.1 |
| DNAAF5 | NP_060272.3 | XP_024213446.1 | NP_001074734.1 | NP_001128329.1 | XP_548719.2 | XP_023510815.1 | XP_010818002.2 | XP_002941855.1 | XP_414755.2 | XP_009302392.3 |
| DNAH1 | NP_056327.4 | XP_016796718.2 | NP_001028840.1 | NP_001028827.2 | XP_022262349.1 | XP_023476087.1 | XP_024839013.1 | XP_012816961.1 | XP_025010454.1 | XP_009304459.3 |
| DNAH11 | NP_001264044.1 | XP_024213530.1 | NP_034190.3 | XP_017458807.1 | XP_022283175.1 | XP_023495115.1 | XP_027395750.1 | XP_017950422.1 | XP_025003205.1 | XP_009290539.1 |
| DNAH5 | NP_001360.1 | XP_016808445.2 | NP_579943.3 | XP_008773325.1 | XP_022269819.1 | XP_023481602.1 | XP_015314607.2 | XP_012820905.1 | XP_025003476.1 | XP_021322524.1 |
| DNAH6 | NP_001361.1 | XP_515578.5 | NP_001158141.1 | XP_017448573.1 | XP_022260075.1 | XP_001916921.2 | XP_024855197.1 | XP_012811375.1 | - | XP_021326862.1 |
| DNAH8 | NP_001193856.1 | XP_016810881.2 | NP_038839.2 | XP_008773178.1 | XP_022281494.1 | XP_023480644.1 | XP_024839646.1 | - | XP_025004702.1 | - |
| DNAI1 | NP_036276.1 | XP_016816249.1 | NP_780347.2 | NP_001019513.1 | XP_022281224.1 | XP_023483256.1 | NP_001033231.1 | - | XP_003643013.1 | - |
| DNAI2 | NP_075462.3 | XP_003953226.2 | NP_001030050.2 | NP_001007727.1 | XP_540403.3 | XP_014594482.1 | XP_002696267.2 | XP_002935429.1 | XP_415701.2 | XP_005156459.1 |
| DRC1 | NP_659475.2 | NP_001233391.1 | NP_001028632.1 | NP_001007010 .1 | XP_532897.3 | XP_014586742.2 | NP_001032680.1 | XP_002938220.2 | XP_420016.3 | NP_001120940.1 |
| HYDIN | NP_001257903.1 | XP_009429461.3 | NP_766504.3 | XP_017443416.1 | XP_022274932.1 | XP_023493062.1 | XP_024834777.1 | XP_012817094.1 | NP_001152843.1 | XP_017207419.1 |
| NME8 | NP_057700.3 | XP_009451181.2 | NP_853622.2 | XP_006254150.1 | XP_013976321.1 | XP_001492951.3 | XP_005205470.1 | - | XP_426021.3 | XP_021334262.1 |
| OFD1 | NP_003602.1 | XP_024209005.1 | NP_803178.2 | NP_001100431.1 | XP_537958.3 | XP_023489673.1 | NP_001179566.2 | XP_002933857.3 | XP_416831.2 | NP_001306137.1 |
| RSPH1 | NP_543136.1 | XP_009434401.3 | NP_079566.1 | NP_001012176.1 | XP_022268910.1 | XP_001491109.1 | XP_005202079.1 | NP_001088789.1 | XP_416745.1 | - |
| RSPH4A | NP_001010892.1 | XP_016811695.1 | NP_001156429.1 | NP_001101099.2 | XP_541210.2 | XP_001504187.1 | XP_002690133.2 | - | - | - |
| RSPH9 | NP_689945.2 | XP_016811076.1 | NP_083614.1 | XP_006244600.1 | XP_005627453.1 | XP_023480767.1 | NP_001039732.1 | NP_001005021.1 | XP_015139198.1 | NP_001025284.1 |
| ZMYND10 | NP_056980.2 | NP_001233432.1 | XP_017168594.1 | NP_001004284.1 | XP_533818.1 | XP_023476145.1 | NP_001035638.1 | XP_018112468.1 | XP_015148255.1 | NP_956691.1 |

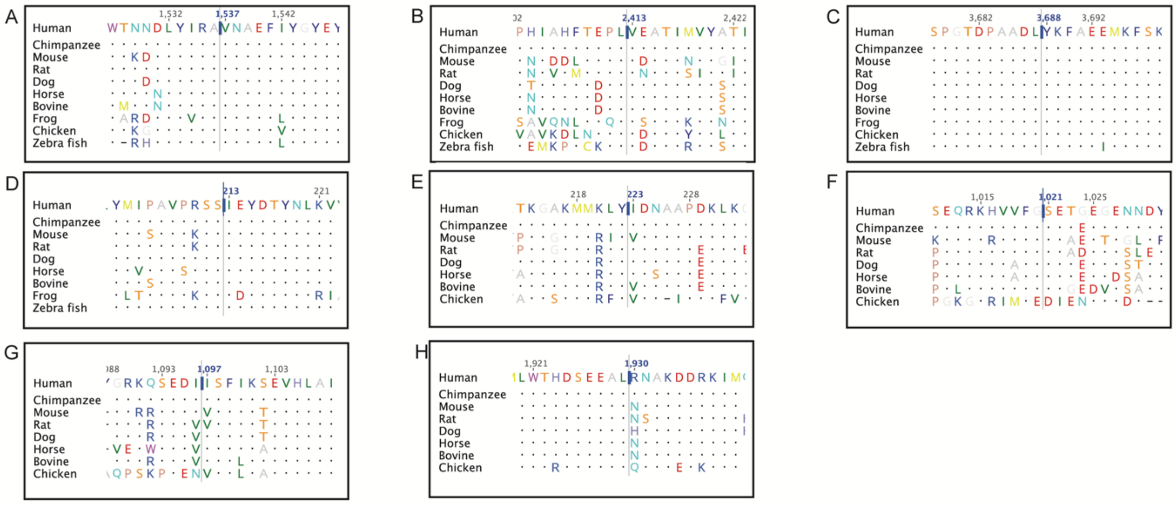

**Figure S1.** Twenty-one amino acid regions, centered around missense/nonsense variations, obtained from a multiple sequence alignment of dynein axonemal heavy chain proteins (other than DNAH5 and DNAH11). DNAH1 protein alignment from human, chimpanzee, mouse, rat, dog, horse, bovine, frog, chicken, and zebra fish are shown in A-C – **A**) V1537M (c.4609G>A); **B**) Vl2413A (c.7238T>C); **C**) Y3688C (c.11063A>G). **D**) DNAH6 protein alignment from human, chimpanzee, mouse, rat, dog, horse, bovine, frog, and zebra fish for I213V (c.637A>G). DNAH8 protein alignment from human, chimpanzee, mouse, rat, dog, horse, bovine, frog, and chicken are shown in E-I – **E**) I223T (c.668T>C); **F**) S1021G (c.3061A>G); **G**) I1097V (c.3289A>G); **H**) R1930H (c.5789G>A).

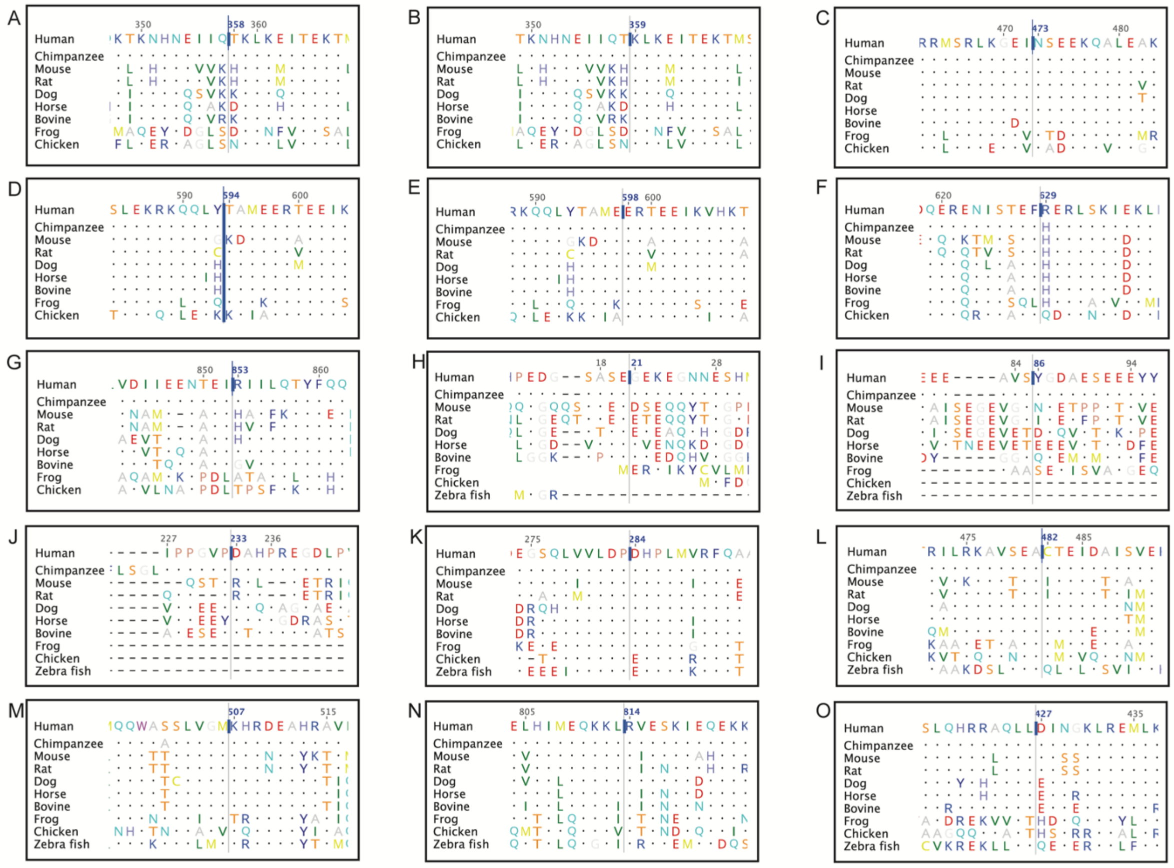

**Figure S2.** Twenty-one amino acid regions, centered around missense/nonsense variations, obtained from a multiple sequence alignment of coiled-coil domain-containing (CCDC) proteins. CCDC39 protein alignment from human, chimpanzee, mouse, rat, dog, horse, bovine, frog, and chicken are shown in A-G – **A**) T358I (c.1073C>T); **B**) K359T (c.1076A>C); **C**) N473D (c.1417A>G); **D**) T594I (c.1781C>T); **E**) E598Ter (c.1792G>T); **F**) R629C (c.1885C>T); **G**) R853C (c.2557C>T). CCDC40 protein alignment from human, chimpanzee, mouse, rat, dog, horse, bovine, frog, chicken, and zebra fish are shown in H-N – **H**) G21V (c.62G>T); **I**) Y86C (c.257A>G); **J**) D233N (c.697G>A); **K**) D284H (c.850G>C); **L**) C482Y (c.1445G>A); **M**) K507M (c.1520A>T); **N**) R814Ter (c.2440C>T). CCDC65 protein alignment from human, chimpanzee, mouse, rat, dog, horse, bovine, frog, chicken, and zebra fish are shown in O – **O**) D427G (c.1280A>G).

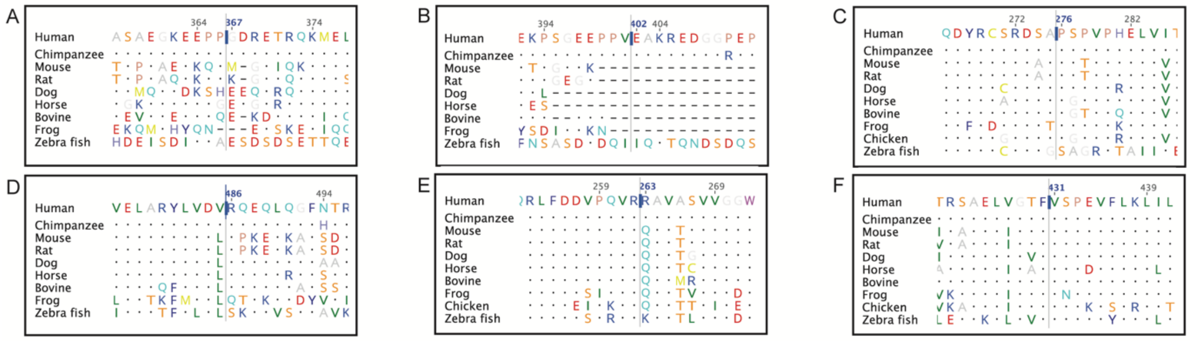

**Figure S3.** Twenty-one amino acid regions, centered around missense/nonsense variations, obtained from a multiple sequence alignment of dynein axonemal assembly factor (DNAAF) proteins. Protein alignment performed using sequences from human, chimpanzee, mouse, rat, dog, horse, bovine, frog, chicken, and zebra fish, where available. **A**) DNAAF1, G367R (c.1099G>A); **B**) DNAAF1, E402V (c.1205A>T); **C**) DNAAF2, P276R (c.827C>G); **D**) DNAAF3, R486W (c.1456C>T); **E**) DNAAF5, R263Q (c.788G>A); **F**) DNAAF5, V431A (c.1292T>C).

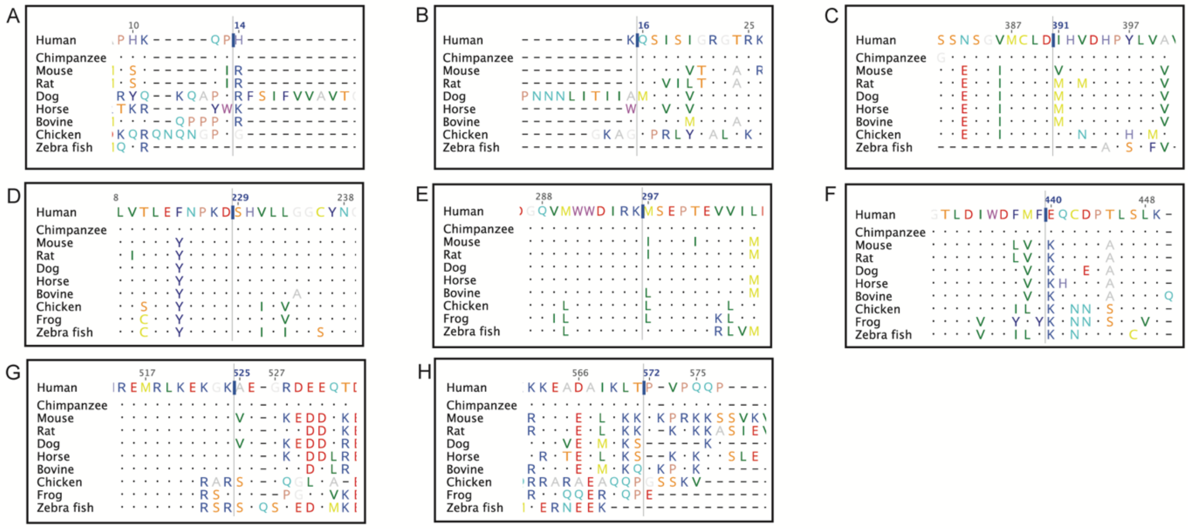


**Figure S4.** Twenty-one amino acid regions, centered around missense/nonsense variations, obtained from a multiple sequence alignment of dynein axonemal intermediate chain (DNAI) proteins. DNAI1 protein alignment from human, chimpanzee, mouse, rat, dog, horse, bovine, chicken, and zebra fish are shown in A-C – **A**) H14Y (c.40C>T); **B**) Q16R (c.47A>G); **C**) I391M (c.1173C>G). DNAI2 protein alignment from human, chimpanzee, mouse, rat, dog, horse, bovine, frog, chicken, and zebra fish are shown in D-H – **D**) S229A (c.685T>G); **E**) M297I (c.891G>A); **F**) E440Q (c.1318G>C); **G**) A525V (c.1574C>T); **H**) P572L (c.1715C>T).

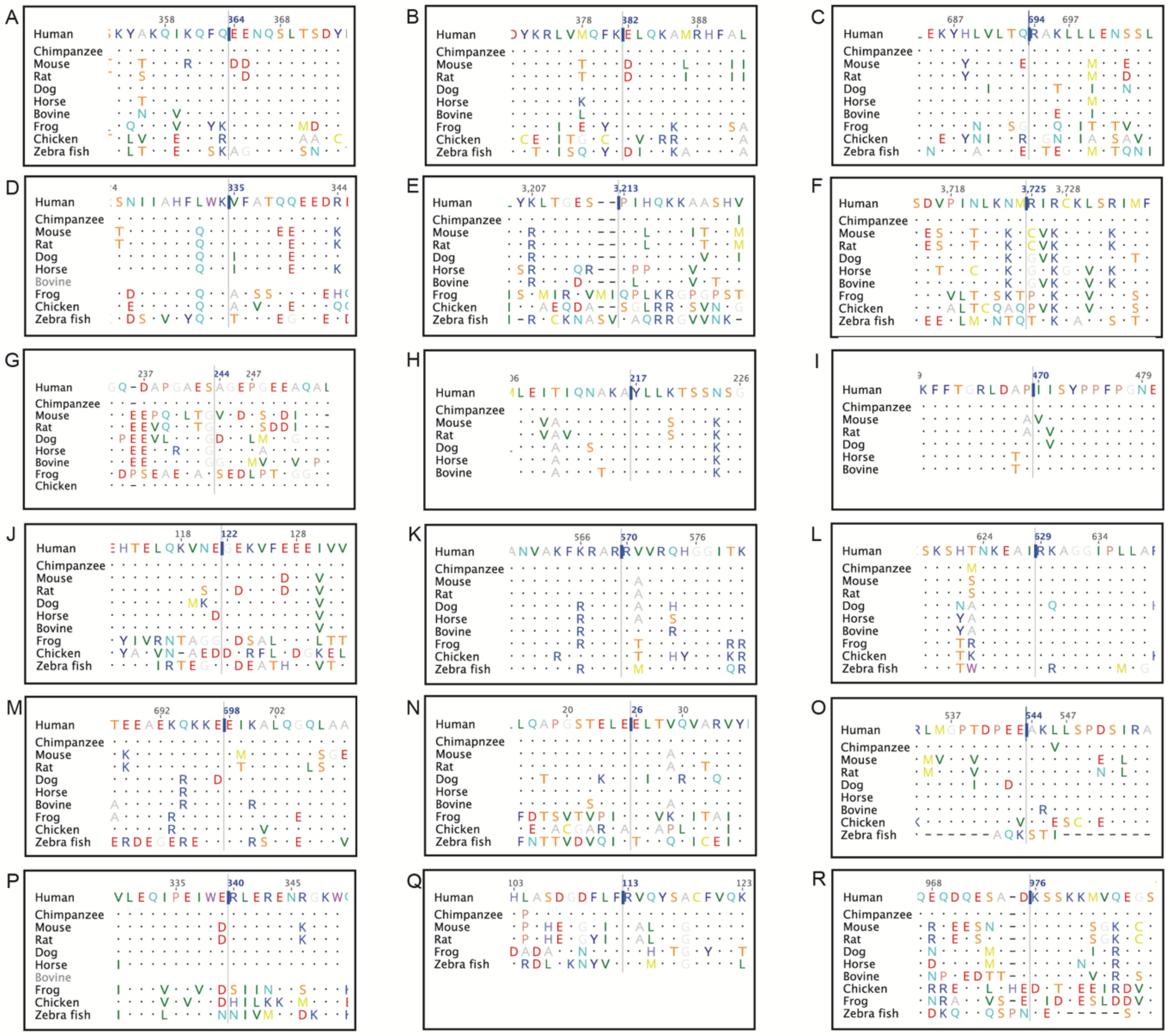

**Figure S5.** Twenty-one amino acid regions, centered around missense/nonsense variations, obtained from a multiple sequence alignment of proteins from human, chimpanzee, mouse, rat, dog, horse, bovine, frog, chicken, and zebra fish, where available. **A**) DRC1, E364K (c.1090G>A); **B**) DRC1, E382D (c.1146G>C); **C**) DRC1, R694T (c.2081G>C); **D**) HYDIN, V335L (c.1003G>T); **E**) HYDIN, P3213R (c.9638C>G); **F**) HYDIN, R3725W (c.11173C>T); **G**) RSPH1, A244T (c.730G>A); **H**) RSPH4A, Y217S (c.650A>C); **I**) RSPH4A, I470M (c.1410C>G); **J**) RSPH9, G122D (c.365G>A); **K**) ARMC4, R570Q (c.1709G>A); **L**) ARMC4, R629H (c.1886G>A); **M**) CEP104, E698K (c.2092G>A); **N**) CFAP298, E26A (c.77A>C); **O**) NME8, A544T (c.1630G>A); **P**) ZYMND10, R340Q (c.1019G>A); **Q**) C1orf127, R113Ter (c.337C>T).
